# Increased aridity drives post‐fire recovery of Mediterranean forests towards open shrublands

**DOI:** 10.1111/nph.16252

**Published:** 2019-11-22

**Authors:** Mara Baudena, Victor M. Santana, M. Jaime Baeza, Susana Bautista, Maarten B. Eppinga, Lia Hemerik, Angeles Garcia Mayor, Francisco Rodriguez, Alejandro Valdecantos, V. Ramon Vallejo, Ana Vasques, Max Rietkerk

**Affiliations:** ^1^ Copernicus Institute of Sustainable Development Utrecht University PO Box 80115 3508 TC Utrecht the Netherlands; ^2^ Department of Evolutionary Biology, Ecology and Environmental Sciences University of Barcelona Av. Diagonal 643 08028 Barcelona Spain; ^3^ Centre for Environmental and Marine Studies Department of Environment and Planning University of Aveiro 3810‐193 Aveiro Portugal; ^4^ CEAM Foundation (Mediterranean Center for Environmental Studies) Parque Tecnológico. C/ Charles Darwin, 14 46980 Paterna Valencia Spain; ^5^ Department of Ecology and IMEM University of Alicante Apdo. 99 03080 Alicante Spain; ^6^ Department of Geography University of Zurich 8057 Zurich Switzerland; ^7^ Wageningen University and Research, Biometris, Mathematical and Statistical Methods PO Box 16 6700AA Wageningen the Netherlands; ^8^ ISEM Université de Montpellier CNRS IRD EPHE 3400 Montpellier France; ^9^ Department of Applied Mathematics and IMEM University of Alicante Apdo. 99 03080 Alicante Spain; ^10^ Erasmus University College Nieuwemarkt 1A 3011 HP Rotterdam the Netherlands

**Keywords:** alternative stable states, climate change, forest fires, increased aridity, Mediterranean shrubland, resprouters, seeders, stochastic dynamical model

## Abstract

- Recent observations suggest that repeated fires could drive Mediterranean forests to shrublands, hosting flammable vegetation that regrows quickly after fire. This feedback supposedly favours shrubland persistence and may be strengthened in the future by predicted increased aridity. An assessment was made of how fires and aridity in combination modulated the dynamics of Mediterranean ecosystems and whether the feedback could be strong enough to maintain shrubland as an alternative stable state to forest.
- A model was developed for vegetation dynamics, including stochastic fires and different plant fire‐responses. Parameters were calibrated using observational data from a period up to 100 yr ago, from 77 sites with and without fires in Southeast Spain and Southern France.
- The forest state was resilient to the separate impact of fires and increased aridity. However, water stress could convert forests into open shrublands by hampering post‐fire recovery, with a possible tipping point at intermediate aridity.
- Projected increases in aridity may reduce the resilience of Mediterranean forests against fires and drive post‐fire ecosystem dynamics toward open shrubland. The main effect of increased aridity is the limitation of post‐fire recovery. Including plant fire‐responses is thus fundamental when modelling the fate of Mediterranean‐type vegetation under climate‐change scenarios.

## Introduction

The extensive land abandonment that occurred worldwide during the last century (Pongratz *et al.*, 2008; Ellenberg & Strutt, 2009) has raised concern about the fate and management needs of old‐field communities (Chazdon, 2008; Cramer *et al.*, 2008). In many different ecosystems, growing evidence supports the possibility of different successional trajectories and the existence of alternative stable states, maintained by internal positive feedbacks (Suding & Hobbs, 2009). Uncertainty about successional trajectories is further enhanced by ongoing climate change, which can modulate ecosystem dynamics and disturbance regimes (Allen *et al.*, 2010; Littell *et al.*, 2016), and may foster novel successional trajectories (Cramer *et al.*, 2008). Understanding how global change drivers and successional dynamics interact and shape future ecosystem states is of utmost importance in order to anticipate the vulnerability and the fate of old‐field communities in a rapidly changing world.

Mature Mediterranean forests mostly comprise broad‐leaved, sclerophyllous species, with a dominance of Holm oak (*Quercus ilex)*, especially in the most mesic conditions (Amici *et al.,* 2013), accompanied by conifers (mostly Aleppo pine, *Pinus halepensis,* and Brutia pine, *Pinus brutia*) under more xeric conditions (Zavala *et al.*, 2000; Zavala & Zea, 2004). The Mediterranean Basin has a millennial history of land use and deforestation, dating back to the Neolithic and peaking in the last two millennia, when a large fraction of the natural vegetation was removed (Vallejo *et al.*, 2006; Connor *et al.*, 2019). However, during the last century, rural depopulation and land abandonment partly reversed these trends, with important consequences for the prevailing fire regimes (Chergui *et al.*, 2018). More specifically, due to land abandonment many old‐field successional communities arose (Hill *et al.*, 2008; San‐Miguel‐Ayanz *et al.*, 2012). The increase in biomass and forest continuity during secondary succession, together with increased aridity due to climate change (Mariotti, 2010; Bedia *et al.*, 2014), has aggravated the fire risk in the Mediterranean in the last decades (Baeza *et al.*, 2006; Santana *et al.*, 2013; Turco *et al.*, 2016).

The classical description of Mediterranean systems proposes that fires simply restart succession by returning the system to an early successional stage (Hanes, 1971; Trabaud, 1984). However, differences in the successional trajectories of old fields with or without the occurrence of fire have been observed (Baeza *et al.*, 2007; Santana *et al.*, 2010). This is particularly evident in the case of short intervals between fires, which may diverge the succession toward a degraded alternative stable state (Baeza *et al.*, 2006; Santana *et al.*, 2010), characterized by a dominance of shrubs and herbs and less fertile soil (Zedler *et al.*, 1983; Haidinger & Keeley, 1993; Lloret & Vilà, 2003; Eugenio & Lloret, 2006; Baeza *et al.*, 2007; Mayor *et al.*, 2016a,b). The persistence of this open shrubland could be maintained by a feedback between fire occurrence and floristic composition, resulting from different fire‐related plant traits and responses (Ackerly, 2004; Odion *et al.*, 2010; Pausas & Keeley, 2014; Batllori *et al.*, 2015, 2019). Early successional shrubs and grasses are more prone to fire than other functional types, due to their high heat of combustion, low water content, fine fuel and capacity to retain standing dead biomass (De Luis *et al.*, 2004; Baeza *et al.*, 2006, 2011; Pausas & Moreira, 2012; Nolan *et al.*, 2018). Furthermore, early successional species can regenerate and spread very rapidly after fire, through different post‐fire responses (Grigulis *et al.*, 2005; Santana *et al.*, 2012; Baeza & Santana, 2015; Vidaller *et al.*, 2019). Specifically, shrubs typically are ‘seeders’ that spread rapidly after fire through their large, and often persistent, seed banks. Grasses are ‘resprouters’ that quickly regrow after fire from their underground reserve system (Keeley, 1986). The combination of fast post‐fire responses and high flammability enables shrubs and grasses to maintain the system in a highly fire‐prone condition. Conversely, the late successional trees, mainly oaks, are slow‐growing resprouters (Clarke *et al.*, 2013; Zeppel *et al.*, 2015), and can outcompete pines, shrubs and grasses (Acácio *et al.*, 2007; Vayreda *et al.*, 2016). These resprouter trees promote fires much less, as they accumulate less fine and dead standing fuel, and their understorey is moister and cooler (Puerta‐Piñero *et al.*, 2007; Saura‐Mas *et al.*, 2009; Tinner *et al.*, 2009; Baeza *et al.*, 2011; Azevedo *et al.*, 2013). These contrasting functional responses have been also observed in other Mediterranean‐climate regions (Odion *et al.*, 2010; Pausas & Keeley, 2014).

Fire–vegetation feedbacks can foster drastic changes in floristic composition, flammability and environmental conditions, yielding the notion that these feedbacks may drive alternative stable states under identical climatic conditions. Specifically, vegetation–fire feedbacks have been proposed as a driver of alternate stable states in tropical forests and humid savannas (Langevelde *et al.*, 2003; Hirota *et al.*, 2011; Staver *et al.*, 2011; Higgins & Scheiter, 2012; Staver & Levin, 2012; Accatino & De Michele, 2013, 2016; Dantas *et al.*, 2016; D’Onofrio *et al.*, 2018), boreal (Johnstone *et al.*, 2010; Rogers *et al.*, 2015; Abis & Brovkin, 2019) and temperate forests (Kitzberger *et al.*, 2012, 2016; Tepley *et al.*, 2016). However, other potential drivers of alternate stable states, mostly related to edaphic conditions, also have been proposed (Fletcher *et al.*, 2014; Bowman & Perry, 2017; Veenendaal *et al.*, 2018). The hypothesis that in the Mediterranean Basin forests and open shrublands are alternative stable states is motivated by observations of succession that has stalled in shrublands (Baeza *et al.*, 2006; Acácio *et al.*, 2009; Santana *et al.*, 2010; Acácio & Holmgren, 2014) and of loss of resilience in oak and pine forests after repeated fires (Diaz‐Delgado *et al.*, 2002; Mayor *et al.*, 2016b). Testing this hypothesis is very challenging, however, as the appropriate time frame to study stability of ecosystem states (Schröder *et al.*, 2005; Bestelmeyer *et al.*, 2011; Fukami & Nakajima, 2011) is typically one to two generations of the longest‐lived species (Connell & Sousa, 1983): in these systems, *Q. ilex* can reach 1000 yr of age (Rigo & Caudullo, 2016), whereas the available direct observations span a few decades or a century at most (Capitanio & Carcaillet, 2008; Santana *et al.*, 2010).

The vegetation–fire feedback described will likely be reinforced by projected climatic changes. For the Mediterranean Basin, an increase in aridity is expected, with higher temperature and more frequent and severe droughts (IPCC, 2013). Projected changes occur more rapidly in this area as compared to the global average (Guiot & Cramer, 2016). Drought affects species composition directly, as water stress limits recruitment, survival and growth (Tweddle *et al.*, 2003; Gómez‐Aparicio *et al.*, 2008; Prieto *et al.*, 2009; Moreno *et al.*, 2011; Peñuelas *et al.*, 2018). Plant types with different responses to fires are associated to dissimilar responses to drought. Seeder shrubs are in general less vulnerable to aridity than resprouter species (Lloret *et al.*, 2005; Saura‐Mas *et al.*, 2009; Pausas *et al.*, 2016). During post‐disturbance resprouting in particular, plants are more susceptible to drought‐induced mortality (Oliva *et al.*, 2014; Pratt *et al.*, 2014; Pausas *et al.*, 2016). Because primary productivity in the region is generally moderate to high (Moreno *et al.*, 2013), droughts increase the probability of fire (Turco *et al.*, 2012; Bedia *et al.*, 2014; Karavani *et al.*, 2018), thus influencing species community composition also indirectly. The combination of these direct and indirect effects can have a dramatic impact on species composition and ecosystem functions (Pratt *et al.*, 2014; Enright *et al.*, 2015), and theoretically they may influence the vulnerability of a landscape to fire (Tepley *et al.*, 2018). Sharp vegetation shifts that occurred in the Mediterranean basin in the past have been associated with the same combination of drought and fire (Colombaroli *et al.*, 2007).

The aim of the present study was to assess the dynamics of Mediterranean ecosystems as affected by the impact of fires and increased aridity. This understanding is fundamental for facing ongoing drastic shifts in vegetation structure and for the development of future management strategies. The complex ecosystem dynamics and their long time horizons (several centuries to millennia) make modelling a valuable approach (Estes *et al.*, 2018), as vegetation dynamics can be simulated up to many generations. The present study adopted a modelling approach for plant competition that includes stochastic fires and differential responses to fire for resprouting and seeding plants. The model parameters were calibrated by quantifying competition and plant growth with observational data from old‐field sites where fire did not occur since land abandonment (between a decade and a century ago), and species responses to fire with observational data from sites where fires did occur in the last four decades. The model was analyzed across a wide, realistic range of parameters around the values obtained from calibration, to ensure the general validity of the results for Mediterranean ecosystems (i.e. beyond the sites used for calibration). The main question was whether Mediterranean oak forests will recover (or persist) under the synergic action of climate change and fire, or whether instead these factors will maintain shrublands as an alternative stable state to forests. An assessment was made of long‐term (centuries‐to‐millennia) ecosystem stability and short‐term (decades‐to‐centuries) risk of ecosystem transitions, which are anthropocentrically relevant, given the urgency of ongoing climate change.

## Materials and Methods

### Model description

A model was developed that describes the dynamics of the main plant types of the Western Mediterranean Basin, including their competitive interactions leading to successional dynamics and stochastic fires (cf. Accatino *et al.*, 2010; Baudena *et al.*, 2010). Furthermore, the differential responses to fires of resprouters and seeders were included.

Six plant types were included, representing the following genus or species: evergreen *Quercus* spp., *Pinus halepensis, Rosmarinus officinalis, Ulex parviflorus, Cistus* spp. and *Brachypodium retusum* (see Table 1, and Supporting Information Notes S1)*.* So, these genera or species represent dominant tree, shrub and grass species in the target ecosystems; for example, in the eastern Iberian Peninsula they jointly cover > 80% of the natural areas under dry meso‐Mediterranean conditions (Baeza *et al.*, 2007; Santana *et al.*, 2010). The oaks (*Quercus* spp.) and the grass (*B. retusum*) can resprout after fires, whereas pines and the shrubs regenerate exclusively via seed germination.

**Table 1:** List of plant types in inverse successional order, corresponding to *i* = 1–6 in the equations.

*i*	Plant type (genus or species)	Acronym	Growth form	Fire strategy
1	*Quercus* (*ilex*, *coccifera*)	Q	Tree (or sub‐tree)	Resprouter
2	*Pinus halepensis*	P	Tree	Seeder
3	*Rosmarinus officinalis*	R	Shrub	Seeder
4	*Ulex parviflorus*	U	Shrub	Seeder
5	*Cistus* (mostly *albidus,* with some *monspeliensis* and *clusii)*	C	Shrub	Seeder
6	*Brachypodium retusum*	B	Perennial grass	Resprouter

The model consists of six ordinary differential equations for the variables *b_i_* that describe the proportion of space occupied by a certain plant type *i* (0 ≤ *b_i_* < 1):

(Eqn 1) $$\frac{db_{i}}{dt}=c_{i}b_{i}\left(1-\sum_{j=1}^{i}b_{j}\right)-m_{i}b_{i}-\left(\sum_{j=1}^{i-1}c_{j}b_{j}\right)b_{i}+\alpha_{i}\left(t\right)\left(1-\sum_{j=1}^{6}b_{j}\right),$$

with *t* representing time (yr), and $1-\sum_{j=1}^{6}b_{j}$ representing the proportion of unoccupied space. See Table 2 for the interpretation of model parameters, their values and units. The first three terms on the right‐hand side (r.h.s) of Eqn 1 represent plant dynamics within the ‘competition model’ (Tilman, 1994; see next section). The fourth term on the r.h.s. of Eqn 1 was included to represent the establishment of seeder plants from their seedbanks after fires. Fires occurred randomly as instantaneous events within the continuous‐time model. The spatial domain was qualitatively defined as an area where the seeds of all plant types could disperse homogeneously (of the order of 10^4^ m^2^), meaning that the model does not explicitly consider long‐distance dispersal. The following sections describe first the successional competition model and subsequently how the fire response of plants was included.

**Table 2:** List of symbols, names, values, units, and their source for the parameters and functions used in Eqns 1 and 2.

Symbol	Interpretation	Values in use for							Units	Sources*
		Q (*i* = 1)	P (*i* = 2)		R (*i* = 3)	U (*i* = 4)	C (*i* = 5)	B (*i* = 6)		
*c_i_*	Colonization rate	0.047	0.053		0.045	0.067	0.11	0.22	yr^−1^	*a*
*m_i_*	Mortality rate= 1/average life time	1/400	1/125		1/50	1/25	1/15	1/40	yr^−1^	*b*
*r_i_*	Fraction of space maintained after fire	0.9	0		0	0	0	0.4	–	*c*
*l_i_*	Flammability (i.e. the inverse of fire average return times if entire plot is covered by one plant type)	1/400	1/20		1/15	1/10	1/10	1/10	yr^−1^	*d*
α_i_	Colonization of seeders after fires	0	See Eqn 2					0	yr^−1^	–
γ_i_	Post‐fire seed germination and seedling establishment	–	0.040	0.0016		0.0029	0.00078	–	–	*e*
*S*_i_	Seed production and storage in the seed bank	–	See Notes S1					–	–	–
*C*	Conversion parameter	–	0.014					–	yr^−1^	*f*

Q, *Quercus* spp; P, *Pinus halepensis*; R, *Rosmarinus officinalis*; U, *Ulex parviflorus*; C, *Cistus* spp; B, *Brachypodium retusum.*

* Sources: (a) optimization of the parameters with the successional data (*c*_1–5_) and with fire data (*c*_6_); (b) (Roy & Sonie, 1992; Panaïotis *et al.*, 1997; Pausas, 1999b; Caturla, 2002; Lloret *et al.*, 2003; Baeza *et al.*, 2006; Raevel *et al.*, 2012; Moya‐Delgado, 2017); (c) *r_1,_* expert estimation; *r_6_*, optimized from fire site data. (d) expert estimation. (e) (Daskalakou & Thanos, 1996; Martínez‐Sánchez *et al.*, 1999; Pausas *et al.*, 2003; Santana *et al.*, 2012, 2014); (f) calibration with fire data.

### Competition model

When the α parameters in Eqn 1 are set to zero, plants compete for space (as in Tilman, 1994), implicitly representing the competition for resources (which, in the system herein, is mostly competition for water in early successional stages, and for light in later stages when the canopy closes). The model assumes a hierarchy between plants, from the strongest (oak, *i* = 1) to the weakest (the grass *B. retusum, i* = 6) competitor, corresponding roughly to an inverse successional order (i.e. from late to early; Sheffer, 2012; Amici *et al.*, 2013; Carnicer *et al.*, 2014). The strongest competitor can outcompete all weaker competitors. Yet, the model‐imposed hierarchy does not necessarily lead to a fixed replacement sequence in the succession: coexistence of all plant types is mathematically possible if a competition–colonization trade‐off is present. For example, for equal mortality rates, an inferior competitor will persist if it is sufficiently faster in colonizing new areas (i.e. has larger colonization rate) than its superior competitors (Tilman, 1994).

The *c_i_* parameters in Eqn 1 are the colonization rates (yr^−1^) and represent which proportion of the total space the existing population of plant type *i* can colonize per capita per time unit, representing a combination of the processes of seed production, germination, and establishment. Note that the space plant type *i* can colonize is equal to $\left(1-\sum_{j=1}^{i}b_{j}\right)$: thus, the total amount of space minus the proportion of space currently occupied by the plant type *i* itself or its superior competitors (as indicated by the summation). These parameters were obtained by model calibration (see the ‘Parameter estimation’ section). The *m_i_* parameters are the plant mortality rates (yr^−1^), equal to the inverse of their life span (Table 2).

### Modelling plant post‐fire responses

Plant post‐fire responses were represented differently for resprouters and seeders. After a fire, the cover of seeders was reduced to zero (by setting the fraction of pre‐fire cover surviving, *r_i_*, to zero, i.e. *r_i_* = 0, for *i* = 2–5), to simulate mortality of all individuals. This implicitly assumes that all simulated fires were severe crown‐fires, which are most common in Mediterranean shrublands and woodlands (Keeley *et al.*, 2012). By contrast, the resprouters retained a fraction *r_i_* of their pre‐fire cover. As the resprouting capacity of *Q. ilex* is extremely high, a rather large baseline *r*_1_ (0.9) was assumed. The resprouting capacity of *B. retusum* was obtained via calibration (see the ‘Parameter estimation’ section). Resprouting was modelled as occurring immediately after a fire, which is the case for most resprouting species (Keeley, 1986).

The seeders’ high post‐fire germination ability due to the (aerial or soil) seed bank was included in the last terms on the r.h.s. of Eqn 1 for *i* = 2–5, whereas the term was set to zero for the resprouters (α_1_ = α_6_ = 0). This term was proportional to the proportion of unoccupied of space (left free after a fire). The parameters α_2–5_ were calculated as a function of pre‐fire seed production and storage in the seed bank *S*_i_(*t*) and of post‐fire seed germination and seedling establishment γ_i_:

(Eqn 2) $$\alpha_{i}\left(t\right)=C\frac{\gamma_{i}S_{i}\left(t\right)}{\sum_{i=2}^{5}\gamma_{i}S_{i}\left(t\right)},\text{for}\;i=2-5,$$

where the denominator corrected for competition for available space after a fire, and *C* was a dimension‐conversion parameter (yr^−1^), estimated from model calibration (see the ‘Parameter estimation’ section). The γ_i_ parameters were estimated from available observations. See Table 2 for parameter values and Notes S1 for details.

The dynamics of the seed‐bank *S*_i_ were different for shrub seeders and pines. For pines, the (aerial) seed bank became available after a fire (representing its fall from the canopies to the ground), and the seeds were then viable for *c*. 2 yr (Pausas, 1999a; Climent *et al.*, 2008). The pine seed bank also depended on the age of the pines before the last fire, because pines only produce seeds when mature (with maturity being reached after 10–12 yr, Pausas, 1999a; Climent *et al.*, 2008). For the shrubs, a gradual decay of the seed bank between fires was assumed. No delay in reproduction was included as these shrubs already produce viable seeds in their first or second year (Moya‐Delgado, 2017)*.* See Notes S1 for details, including the mathematical functions representing these processes and their parameter values.

### Stochastic fire occurrence

Fires were modelled as stochastic events with the time between two consecutive fire events being described by an exponential distribution with average time *T*_f_ (yr). Higher cover of the more flammable species (i.e. larger flammability *l*_i_; see Table 2) decreased the average fire return time (D’Odorico *et al.*, 2006; Baudena *et al.*, 2010):

(Eqn 3) $$T_{f}={\left(\sum_{i=1}^{6}l_{i}b_{i}+\varepsilon \right)}^{-1}.$$

The term ε assured that when total plant cover was zero the fire return time would be very large (1/ε = 10^4^ yr) but not infinite, to avoid numerical instabilities. A minimum of 2 yr was set for fire return time.

### Dataset description

#### Old‐field data

**Figure 1:**
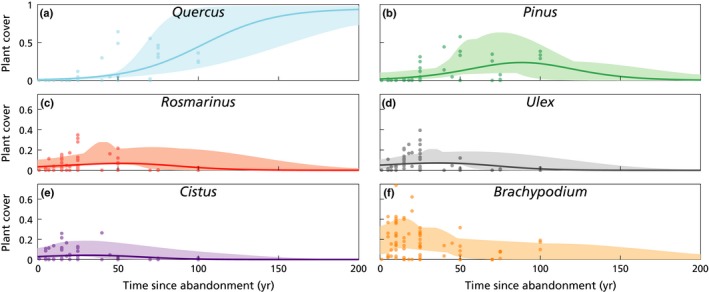
Plant cover of the old‐field data (symbols) and of the competition model runs (lines) for the six plant types, as a function of the time since land abandonment. Model trajectories were obtained with colonization parameters *c*_1–5_ as in Table 2, which correspond to the best fit obtained by calibration with the old‐field data shown (*H* ^2^ = 0.70). The model trajectory for the grass (*Brachypodium retusum*, panel (f)) was omitted because *c*_6_ was calibrated using the fire data. Shaded areas indicate the extent of all possible trajectories as obtained with Monte Carlo variations within the calibration procedure (see Supporting Information Notes S1.4).

In order to calibrate the competition model, data were used from different sites where plant cover had been recorded in old fields, ranging from 1 to 100 yr since abandonment, and where no fire had occurred since (‘old‐field data’). A total of 73 sampling sites were selected from previous studies located in the Western Mediterranean Basin in Eastern Spain and Southern France (Rodriguez‐Aizpeolea *et al.*, 1991; Tatoni, 1992; Padilla, 1997; Peña, 2007; Santana *et al.*, 2010). Vegetation in these sites was mostly composed of the six plant types described above, generally not overlapping in space (Notes S1). All sites are characterized by a dry meso‐Mediterranean climate (mean annual precipitation: 480–814 mm yr^−1^, mean annual temperature 14–17°C, aridity index (UNEP, 1992): 0.62–0.96), are located over basic bedrock type (marls or limestones), and represent the actual range of environmental conditions for the selected plant types in their natural distribution area within fire‐prone Mediterranean ecosystems (Table S2 in Notes S1). Space‐for‐time substitution was used and these data treated as a time series (Blois *et al.*, 2013) of plant cover since land abandonment (Fig. 1).

#### Fire data

In order to calibrate the fire model, a time series was used of plant cover from four experimental sites located in SE Spain (‘fire data’). These sites are part of a permanent long‐term study established in 1994 (Santana *et al.*, 2013). Each of them had experienced one wildfire during the last three decades, and some plots within the sites were burned once or twice experimentally. The regularly sampled data from the experimentally burned plots were used to define four temporal series of plant cover (Fig. S2; Table S3 in Notes S1).

### Parameter estimation

The model parameters *c*_1–6_, *r*_6_ and *C* were calibrated with the two sets of data, obtaining a standard parameter set (Tables 2, S1 in Notes S1), used throughout the study, unless specified otherwise. In the calibration, the parameters were determined as those minimizing the mean square deviation between modelled time‐trajectories and data (with respect to the variance of the data; Baudena *et al.*, 2012). As such minimization is nontrivial, requiring a large number of model runs to cover the multidimensional parameter space, the simulated annealing optimization algorithm (Kirkpatrick *et al.*, 1983; see Notes S1 for a detailed description) was adopted.

Optimal values for the colonization rates *c*_1–5_ were obtained by calibrating the competition model with the old‐field data (‘old‐field‐cal’); see the resulting model trajectories in Fig. 1(a–e). For *c*_6_, the value estimated from the fire data (see subsequent paragraph) was preferred, because the old‐field‐cal determined *c*_6_ with a very large error.

The complete model was calibrated using the fire data to obtain optimal values of the three parameters *C*, *r*_6_ and *c*_6_ (chosen as they showed the greatest improvement in the goodness‐of‐fit, see Notes S1). See Fig. S2 in Notes S1 for the model trajectories and the fire data.

A range of realistic values also was estimated for the colonization rates, with which the general validity of the results herein could be shown beyond the specific values of the standard set. For this, the old‐field‐cal was repeated taking into account the uncertainties in the data (e.g. due to the space‐for‐time substitution) with a Monte Carlo approach (MC) (Jakoby *et al.*, 2014; D’Onofrio *et al.*, 2015). The resulting variation in the model trajectories thus obtained is represented by the shaded areas in Fig. 1; see Notes S1 for details.

### Analyses

An analysis was undertaken for whether Mediterranean oak forest would regrow or persist simulating historical and increased aridity conditions. Within each simulation, climatic conditions were constant (with the exception of one experiment, see Short‐term experiments and Notes S5.2). Climate was implicitly implemented via its effects on the plant parameter values. Experiments were performed at two different timescales: (1) a ‘long‐term’ (centuries‐to‐millennia) scale, characteristic of the ecosystem dynamics, and (2) a ‘short‐term’ (decades‐to‐centuries) scale, characteristic for anthropogenic impact management. The model was solved numerically (Fortran code, Runge–Kutta integration with time steps of 1/365 yr). A few analytical calculations were performed to reinforce the numerical results, obtaining analytical values for the oak cover within a simplified version of the model, where fires had an imposed frequency, independent of species composition (Notes S2).

#### Historical and increased aridity

Three different effects of historical and increased aridity were included, modifying different parameter values in a full‐factorial design:

1. oak resprouting ability after fires decreasing with water stress (Galiano *et al.*, 2012; Pausas *et al.*, 2016). The oak parameter *r*_1_ was lowered from 0.9 to 0.75 and 0.6; it was not decreased to smaller values because the oak target species are extremely good resprouters in any condition (Espelta, Retana & Habrouk, 2003).
2. lowering reproduction and establishment, represented by colonization abilities *c*_i_. Water stress is expected to influence oak growth, reproduction and establishment negatively (Ogaya & Peñuelas, 2007; Peñuelas *et al.*, 2018), and possibly to a larger extent than for the other plant types (Peñuelas *et al.*, 2001; García‐Valdés *et al.*, 2015; Pausas *et al.*, 2016). Thus, the effect of a decrease in the colonization rate of oaks was first analyzed, with *c*_1_ lowered between 0.047 and 0.011 yr^−1^, without affecting the colonization rates of the other plant types. To simulate the predicted severe changes in aridity in the Mediterranean area, this reduction was well beyond the range of variation of the parameters as detected in several ways by the old‐field‐cal (see Notes S1.4), which represented plant colonization under historical climate conditions.
3. increasing the flammability *l*_i_ of all plant types (between the original values, as in Table 2, and three‐fold higher values), to represent the increased fire ignition and rate of spread due to decreased fuel moisture content (Nolan *et al.*, 2016, 2018; Karavani *et al.*, 2018).

Thus, there were 48 sets of parameters (3 *r*_1_ values × 4 flammability values × 4 *c*_1_ values), including the baseline aridity scenario representing historical climate conditions (as given by the standard set). Each parameter set was used in runs with different initial conditions, and for both timescales described below. Furthermore, for the long‐term experiments, it was analyzed what would happen if all six plant types were affected by aridity, with specific runs and by varying all colonization rates with a Monte Carlo approach (see end of Long‐term experiments section and Notes S4 for details). In one of the two short‐term experiments, aridity was not constant but increased over time (see Short‐term experiments and Notes S5).

#### Different timescales

##### (1) Long‐term experiments

**Figure 2:**
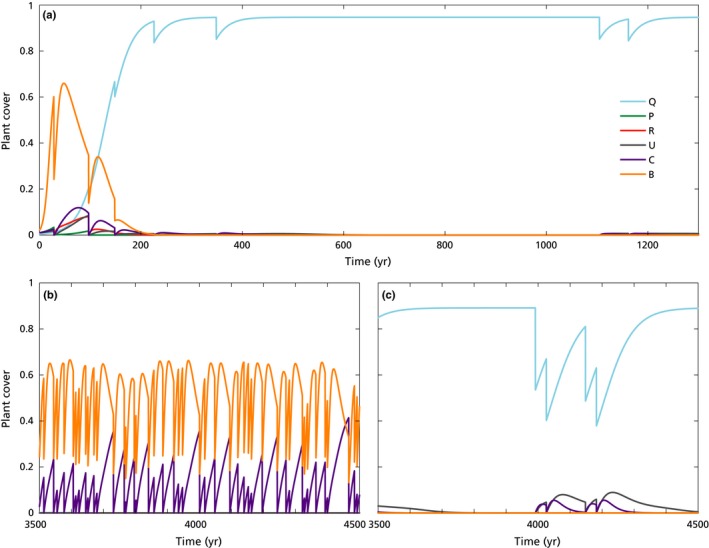
Plant cover as a function of time for the six plant types (long‐term simulations). Each discontinuity in the lines indicate that a fire occurred, with frequency that depends on plant community composition. (a) Current climate conditions: after a transient period where all the plant types co‐occur, the system converged to an oak forest. The specific details of the first part of the trajectories depended on the initial conditions and on the stochastic fire sequence (here *b*_0,1–6_ = [0.0039, 0.01, 0.01, 0.01, 0.01, 0.02]). Average fire return time when oak established was *c*. 275 yr (calculated between 200 and 1300 yr, as shown here for clarity of visualization). (b, c) Increased aridity conditions lead to (b) open shrubland and (c) oak forest (*r*_1_ = 0.60, *c*_1_ = 0.0023 yr^−1^, and flammability 1.2‐fold the baseline value, given in Table 2; marked as bistable in Fig. 3c). Not only the plant cover, but also the emergent fire frequencies were different for the two systems: every *c*. 500 yr for the oak forest, every *c*. 27 yr for the open shrubland (calculated on the last 2000 yr of the simulation). For clarity of representation, only a part of the 10 000 yr‐long simulation is displayed here. See legend in (a) for colour codes and Table 1 for plant acronyms; parameters not mentioned here are as in Tables 2 and S1 (Supporting Information Notes S1).

**Figure 3:**
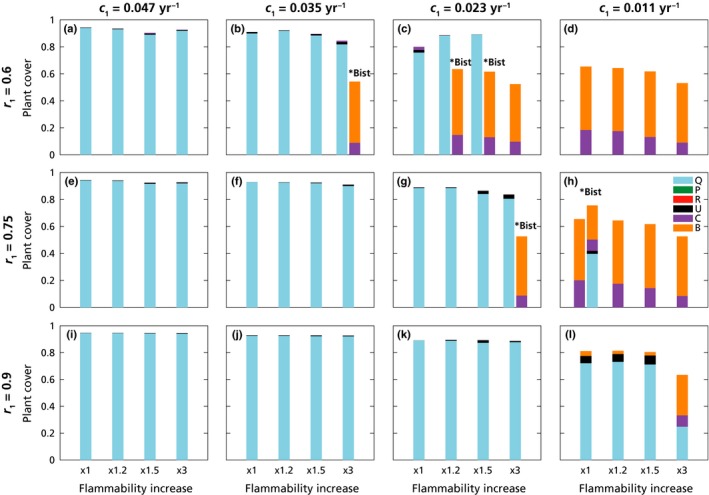
Plant composition under the 48 aridity scenarios in the long‐term experiments. Bars represent average plant cover (calculated between 8000 and 10 000 yr from start of run). Aridity increased from current level (leftmost bar in panel (i)), affecting three aspects : (1) *x*‐axis, left to right: increasing flammability (between one‐ and three‐fold the baseline values of Table 2); (2) from left to right panels: decreasing values of oak colonization ability *c*_1_; (3) from bottom to top panels: decreasing values of oak resprouting ability *r*_1_. The top‐right panels (c, d, g, h) and the rightmost bars represent the harshest aridity conditions. Simulations with two bars and with the label ‘*Bist’ (in b, c, g, h), represent the plant composition of the two alternative stochastically stable state. See legend in (h) for colour code, and Tables 2 and S1 in Supporting Information Notes S1 for parameter values.

In order to represent the long‐term ecosystem dynamics, each model run simulated 10 000 yr. These long runs also assured a good statistical representation of the plant cover oscillations due to the stochastic fire perturbations (Fig. 2). Values of final plant cover were calculated as the average cover during the last 20% of each model run (e.g. Fig. 3). If fires were not included, model runs simulated 1000 yr.

For each of the 48 parameter sets described above, representing historical and increased aridity conditions (Fig. 3), the simulations were repeated for six different initial conditions (Table S8 in Notes S1). Furthermore, to explore the effect of the fire‐vegetation feedback, an extensive MC analysis was performed to test for path‐dependency on plant cover initial conditions, including 4010 sets of random initial conditions for each of the 48 parameter sets. From these runs the time to oak dominance also was determined, a parameter which characterized the forest (re)growth (see Notes S3).

Another MC analysis (more than 180 000 simulations in total) also was performed, where the colonization rates *c_i_* of all plant types were varied over a broad range of values (Table S10 in Notes S4), with three aims: (1) including the effects of aridity on all plant types (instead of only affecting oaks); (2) assessing the validity of the results herein beyond the specific sets of 48 simulations; (3) including parts of the parameter space where pines and oaks could theoretically coexist in the absence of fire. See Notes S4 for further details.

##### (2) Short‐term experiments

**Figure 4:**
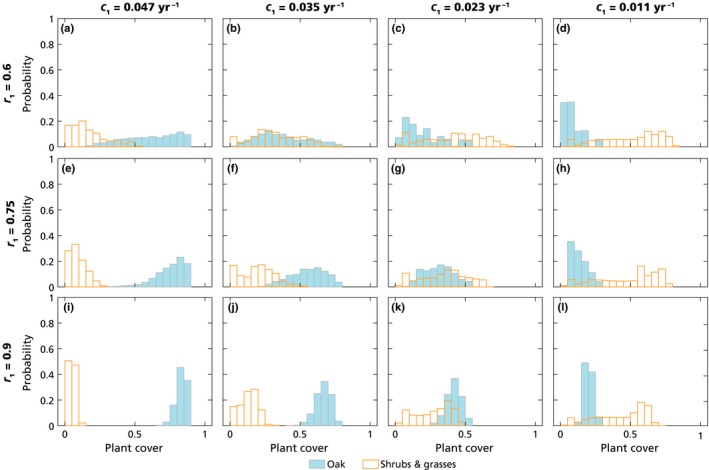
Probability distribution of oak cover (filled blue bars) and shrubs + grass cover (open yellow bars) in the short‐term runs, calculated between 80 and 100 yr after the beginning of the simulation and across the 100 runs, for 12 combinations of the parameters *r*_1_ and *c*_1_, representing harsher aridity conditions when moving towards the right and upward in the figure (i.e. panel (i) represents the lowest aridity level and panel (d) the highest aridity level). The system was initialized with a mixed successional community with equal cover of all the plant types (Table S12 in Supporting Information Notes S5). Flammability was 1.5‐fold the baseline value; other parameters are as in Tables 2 and S1 (Notes S1).

In order to predict the response of the Mediterranean ecosystems in the anthropogenic climate change context, the dynamics were analyzed over a climatically relevant timescale of 100 yr. For this timescale, a probabilistic approach was used, running 100 simulations for each of the 48 parameter sets, representing different aridity conditions. The probabilistic approach across runs was necessary given the stochastic fires, because at this short timescale the system states depended on the specific fire sequence realized in a run. The system was initialized with three contrasting initial conditions representing different present‐state communities: oak forest, shrubland with grasses and a mixed ‘successional community’ (including 15% cover for each of the plant types; Table S12 in Notes S5). To characterize community composition at this timescale, the probability distribution of oak cover and of the sum of shrub and grass cover in the last 20 yr of the runs were analyzed, across all of the runs performed with a specific aridity parameter set and initial conditions (Fig. 4). Probabilites also were quantified of oak forests decaying by the end of the century (*b*_1_ < 50% or 65% cover), or growing > 30% cover if starting from shrubland or mixed successional communities (Table S13 in Notes S5).

Finally, it was verified whether the predictions for oak forest recovery or persistence of the short‐term experiments would change substantially if aridity harshened over time (see Notes S5.2).

### Data availability

Model scripts and field data are available on the platform github.com at: https://github.com/baudenam/FireMed-Baudena-et-al-2019-New-Phytologist.

## Results

### Vegetation dynamics under historical climate conditions

Although coexistence is theoretically possible in the competition model without fires (Tilman, 1994), long‐term coexistence of the plant types defined herein was not observed. Instead, the oak became dominant (here defined as *b*_1_ > 0.5) between 75 and 150 yr after abandonment, and all other species disappeared within the first 150–200 yr (Fig. 1). Oak dominance was achieved for a wide range of parameter values; that is, this result was not sensitive to variations of the model parameters within their margins of uncertainty (shaded areas in Fig. 1, as obtained with the old‐field‐cal; see Notes S1 for details).

When fires were included, plant cover values did not attain equilibria but kept varying as a consequence of the resulting stochastic disturbances. Despite these fire disturbances, the oaks dominated the system in the long term, under any initial condition and fire frequencies, given enough time to establish, when using the standard set of parameters (Figs 2a, 3i leftmost bar). The time at which oaks became dominant varied widely (up to 300 yr after abandonment), depending on the initial cover of oak, but not on the initial cover of the other plant types (see details in Notes S3). Fire return time was *c*. 275 yr when oak dominance was achieved (calculated between 200 and 1300 yr since land abandonment in Fig. 2a). Not surprisingly, in the short‐term experiments the plant composition varied depending on the initial cover of the different plant types (Notes S5), as the short‐term runs lasted less than the 400‐yr average life span of oaks in the present model. The communities displayed a general tendency toward increase of oak cover. Forest always persisted, whereas a system initially dominated by shrubs and grasses experienced substantial increases in oak cover within the century, reaching values > 0.3 with a 46% probability. A mixed successional community always developed into a forest with oak cover always > 70% (Table S13 in Notes S5). Pines would persist until two subsequent fires occurred close enough to each other (not shown), as no pine reached seed‐production maturity (Thanos & Daskalakou, 2000).

### The effect of fires and increased aridity

**Figure 5:**
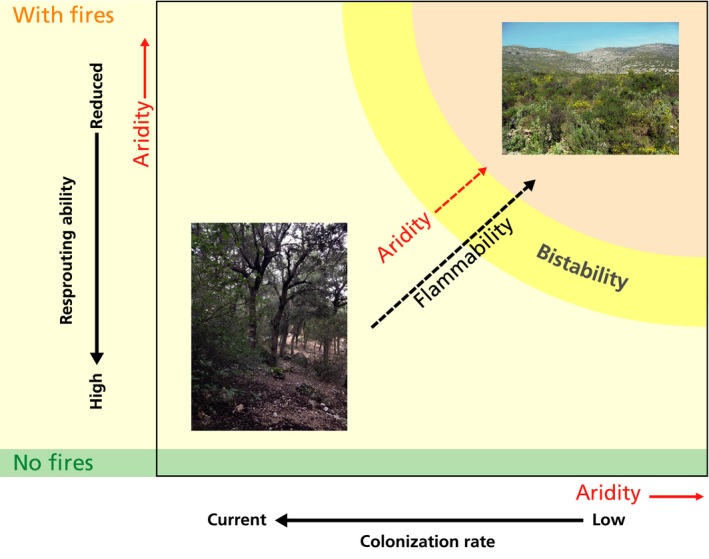
Conceptual scheme of the long‐term model results. The combined action of aridity and fires led the system to an open shrubland (top‐right) instead of an oak forest (bottom‐left). Aridity acted along three different axes: it decreased colonization (*x*‐axis) and resprouting ability (*y*‐axis) after fires, resulting in an open shrubland instead of a closed oak forest, and it also impacted flammability (along the diagonal). Only two of the three types of effects were necessary to observe the transition between the states. Stochastic bistability between forest and shrubland was observed in an intermediate region, if flammability was increased increased at least a little (see also Fig. 3).

When simulating the effect of increased aridity on plant community composition, it was observed that a large increase in aridity could lead to a very different community in the long‐term runs, namely an ‘open shrubland’, composed of *Cistus* and *Brachypodium*, with a lot of space left unoccupied (> 50% in some cases; Fig. 3). The open shrubland is a stochastically stable state; that is, maintained by the stochastic fires. The open shrubland appeared because of the combined effects of increased aridity on the colonization and resprouting capacity of oaks and on fire return times (Fig. 3). By contrast, if aridity decreased only oak colonization ability, but no fires occurred, the model would converge to an oak forest for all considered aridity levels. The only effect of aridity in the absence of fire would be a reduction in oak cover (from *c*. 0.90 to 0.77), with grasses coexisting with oaks at the strongest aridity level considered (not shown; see illustration in Fig. 5). This dependence was supported by analytical calculations using a simplified version of the model (see Notes S2 for details).

For the open shrubland state to occur, aridity needed to affect at least two different factors, (e.g. reduction of the resprouting ability and the colonization capacity of oaks) (Fig. 3). Notice that the open shrubland was observed independently of the assumption of a relationship between fire frequency and aridity. Specifically, fire frequency was not imposed but emerged from the plant community composition and cover (average return time between *c*. 150 and 2000 yr for the forest, and 13–33 yr for the open shrubland). Modelled aridity increased the flammability of all plant types but not necessarily the fire frequency, because the latter depended also on the plant cover. The open shrubland also was observed in some cases without increases in flammability, if aridity diminished oak colonization and resprouting abilities (Figs 3d,h). Finally, the results did not change in any major way when aridity was assumed to affect all plant types and not only the oaks (see Notes S4).

Alternative stable states between a forest with nonfrequent fires or an open shrubland with frequent fires were observed (see Fig. 2b–c for two example time series). Alternative stable states occurred in that part of the parameter space representing intermediate conditions, that is with either maximal increase in flammability and intermediate decreases of colonization rates and resprouting capacity, or with intermediate increases in flammability but strong reduction of resprouting or colonization capacity (Fig. 3). Technically, this was a case of ‘stochastic bistability’: stochastic perturbations acting on a dynamic system give stability to an otherwise unstable state (Kapitza, 1951). In the present case, the latter state would be the open shrubland, whereas the other stable state (i.e. the forest) would be the only stable state of the system without stochasticity. By means of the MC analysis on initial conditions, given an initial species composition, it was not possible to predict the final state of the system unequivocally (see Notes S3). The stochasticity in the system thus overwhelmed most of the effects of the initial conditions, so that even oak forests had a finite chance to convert into open shrublands at these intermediate aridity levels, and a shrubland might or might not develop into a closed oak forest. Generally, the probability of the forest as a final state increased with the initial oak cover and decreased with the initial cover of shrubs and grasses (Figs S6–S8 in Notes S3).

Finally, the MC analyses with varying colonization rates showed that the long‐term results were not dependent on the specific values used in the 48 parameter sets composed, but were valid across a realistic range of parameters. With these MC simulations it also was observed that pines could survive in the long‐term experiments even in the presence of fires, together with oaks or in a shrubland, in parts of the parameter space where oaks and pines could coexist in the competition model (without fires). Pine survival was only observed in a small portion of the MC runs in this part of the parameter space, and only if flammability was not substantially increased, probably due to the higher chance of two fires occurring too close after each other at higher flammability values, preventing pines to reach maturity. See Notes S4.2 for details.

Aridity increased the tendency to shift toward shrubland, which could already occur over the short‐term scale considered. The probability of a mixed successional community becoming an oak forest after 100 yr decreased drastically with increasing aridity (moving from bottom left to top right in Fig. 4, for an example with flammability equal to 1.5 times the baseline value). Differently from what was observed for the current aridity levels, oaks had no chance to establish in a shrubland when aridity was high or medium‐high (Fig. S14 in Notes S5). Even oak forest persistence was compromised by aridity increases (Fig. S13), with up to 15% chance of oak cover decreasing below 0.5, reaching as low as 0.15 in some runs (Table S13). Similar conclusions could be drawn from model runs in which aridity gradually increased during the 100‐yr simulations (see Notes S5 for further details).

## Discussion

The combined effect of competition and fire dynamics in the Mediterranean Basin under historical climatic conditions, even though perturbed by frequent fires, led to a dominance of the late‐successional oaks on the long term (millennia) in the present model, involving a process of canopy closure that could already be observed on the short term (decades‐to‐century). However, the present model predicted that future potential increases in aridity may drive these fire‐prone ecosystems past a tipping point, after which open shrublands stably replace closed forest.

The resilience of oak forests under historical conditions could be attributed to their high post‐fire resprouting capacity, relative low flammability and ability to outcompete other species (mostly by shading) in the long run. This result is in line with palaeo‐ecological assessments reporting that current open shrubland landscapes emerged as a consequence of human activity during the past 2000 yr (Colombaroli *et al.*, 2007; Tinner *et al.*, 2009), and that forest landscapes could persist under current climatic conditions (Henne *et al.*, 2013, 2015; Tinner *et al.*, 2016). The long‐term simulation results herein and palaeo‐ecological analyses span at least a few millennia. Going well beyond the time span covered by direct observations, this time frame is needed to draw conclusions about succession between ecosystem states that are dominated by long‐lived species.

Despite these inherent long‐term characteristics, the resilience of the oak forest was already apparent over the 100‐yr timescale: oak forests persisted under current conditions (Fig. S13 in Notes S5), whereas mixed successional communities (Fig. 4) and shrublands (Fig. S14 in Notes S5) tended to experience an increase in oak cover. Although long‐term simulations all converged to either a forest or a shrubland (Fig. 3), short‐term simulations were technically ‘transient’, showing large variability in plant composition between simulations, mainly depending on initial conditions and the specific stochastic fire sequence realized in each simulation. In many short‐term simulations, communities included pines, shrubs and grasses, with oaks comprising only a minor proportion (e.g. first century in Fig. 2a; Fig. S14). The mixed temporary communities could persist for a variable amount of time, partly depending on the initial oak cover (Notes S3), where low cover values could be interpreted as a proxy of oak seed availability and recruitment limitation (Sheffer, 2012). The multiscale approach used herein provided an explanation of the contrasting findings within direct observations and other (short‐term) modelling predictions, which are bound to show different results depending on the local history (e.g. of land use). In old fields, assemblies of shrubs and pines without oaks prevail under the current fire regime (Lloret *et al.*, 2003; Pausas & Lloret, 2007; López‐Poma *et al.*, 2014), with pine disappearing under repeated fires (Daskalakou & Thanos, 1996; Eugenio & Lloret, 2004). However, direct observations also show that fires can transform pine forests into oak forests if the latter are present in the understory (Retana *et al.*, 2002; Torres *et al.*, 2016; Vayreda *et al.*, 2016; Martín‐Alcón & Coll, 2016). The present model results also reinforced state‐of‐the‐art restoration findings, showing that the planting of resprouting oaks in shrublands significantly redirects and accelerates the transition towards late‐successional oak communities (Santana *et al.*, 2018).

Mediterranean vegetation is threatened by the expected increase in aridity due to climate change (IPCC, 2013; Guiot & Cramer, 2016; Turco *et al.*, 2018). It was shown that increased aridity could disrupt the resilience of oak forests. When fires occur, water stress is expected to reduce the post‐fire resprouting capacity of oaks because of, for example, higher mortality and water‐stress induced cavitation (Cruz & Moreno, 2001; Vilagrosa *et al.*, 2014; Pratt *et al.*, 2014; Pausas *et al.*, 2016). Water stress alone, reducing plant growth and seedling establishment for all species, and especially oaks (Ogaya & Peñuelas, 2007; Gómez‐Aparicio *et al.*, 2008, 2011; Ruiz‐Benito *et al.*, 2012), would not hinder the development of old‐field communities and shrubland into forests in the present model. Yet, the combination of these effects of water stress with limited post‐fire recovery, could drive the old‐field communities towards a shrubland state. At very high levels of aridity, even established forests would not persist and be replaced by open shrublands, the only stable state. At intermediate aridity, forests might already shift to shrublands, as the two are alternative stochastically stable states (D’Odorico *et al.*, 2006; Beckage *et al.*, 2011). This is expected in systems where flammability declines during succession (Kitzberger *et al.*, 2012), and it follows from the interaction between decreased post‐fire oak recovery rates and the positive feedback driven by the high shrubland flammability and fast recolonization after fires (similar to the scenario shown by Tepley *et al.*, 2018, for temperate forests). If the effects of water stress on post‐fire responses are not included, modelling efforts might overestimate forest resilience (Henne *et al.*, 2015). The results herein further underpins the recent findings of Batllori *et al.* (2017, 2019), who showed with a theoretical model for Mediterranean systems, that shrublands would expand at the expense of forest under high drought recurrence combined with fire, although only for certain parameter values.

A choice was made to represent competition indirectly (following, e.g., Hastings, 1980; Tilman, 1994; Staver & Levin, 2012; Abis & Brovkin, 2019), with a parsimonious approach that allowed the clear identification of the importance of aridity‐driven decreases in post‐fire responses, and to support this finding with analytical calculations. The approach was not immune to shortcomings. First, aridity was included implicitly in its effect on vegetation, thus allowing for investigating the effect of average harshening due to expected strong increases in mean annual temperature and decreases in annual precipitation in the area (Guiot & Cramer, 2016). However, the changes in temporal rainfall distribution, for example, were not investigated, which also are expected to be dramatic (Giorgi, 2006). Secondly, at the highest aridity level, an open shrubland was obtained where fires still occur. This is a good representation for mesic Mediterranean regions, where fires are less fuel‐limited than drought‐limited (Pausas & Paula, 2012; Turco *et al.*, 2017), and the space between living resprouter plants is often occupied by fine standing dead woody biomass, mostly from seeders, which is very important for fire spread (Baeza *et al.*, 2002; Baeza & Santana, 2015). It must be acknowledged that this effect will however not increase indefinitely, as further aridity increases also would decrease fuel connectivity. To include this, further analyses could represent fire frequency as decreasing nonlinearly with cover (Accatino *et al.*, 2016; Yatat *et al.*, 2017). The implicit space approach used herein did not represent spatial processes at the landscape scale, such as distance to seed sources, seed dispersal ability and fire spread. A model extension including spatially contiguous cells could for example verify whether the alternative shrubland and forest states result in patchy landscapes, or whether the contiguous presence of forests and shrublands would actually facilitate landscape‐scale changes towards one or the other state (Kitzberger *et al.*, 2012; Schertzer & Staver, 2018; Li *et al.*, 2019). The CO_2_ fertilization effect, acting in parallel to aridity intensification, is another element that will affect future vegetation in the area, although its effects are still debated (Keenan *et al.*, 2011). Finally, adding more plant responses and types in models has the well‐known downside of exponentially increasing the number of parameters. Although the number of variables does not necessarily limit analytical tractability (Eppinga *et al.*, 2018), it becomes generally more challenging to determine the parameter values. To the best of the present authors’ knowledge, this issue was tackled by an extensive model calibration.

Oaks and pines often co‐occur in Mediterranean forests and a large body of literature has tried to explain this association (Zavala & Zea, 2004; Gómez‐Aparicio *et al.*, 2011; Zavala *et al.*, 2011; Sheffer, 2012; García‐Valdés *et al.*, 2015). Generally, pines replace oaks with increasing aridity conditions (Sheffer, 2012). The present model showed that, as expected, forests with pines and oaks were possible when including the improved performance of pines with increased aridity (by increasing their colonization rate; Notes S4). However, the mechanisms that mediate pine–oak competition for light and water are more complex than classically believed, with shifts in the competition between different tree life stages (Zavala *et al.*, 2011) that were not included in the present model. This approach was sufficiently accurate as it was found that the main limitation to pine persistence was fire recurrence and associated post‐fire availability of pine seeds (Thanos & Daskalakou, 2000; Baeza *et al.*, 2007; Tucker & Cadotte, 2013; López‐Poma *et al.*, 2014): in the present model, pines could not survive even when they were competitively favoured over oaks, if two fires would occur too close to each other (i.e. < *c.* 10 yr; Notes S4).

Both globally and in the Iberian Peninsula, a hump‐shaped relationship between fire and productivity (or aridity) has been identified and explained according to the interplay between different mechanisms. An increase in aridity in temperate regions can lead to increased frequency of fire‐prone conditions, for instance due to decreased vegetation moisture, whereas in the most arid areas fire frequency is decreased by the low productivity and the loss of vegetation connectivity (Pausas & Paula, 2012; Mcwethy *et al.*, 2013; Pausas & Ribeiro, 2013). For these reasons, the connection between increased aridity and fire frequency can change, for example in different areas in the Mediterranean, or as a consequence of fire prevention strategies (Turco *et al.*, 2016, 2017). In the present model, these two counteracting mechanisms were included directly, and not the hump‐shaped fire–aridity relationship. A modelled increase in aridity rendered plants more flammable, but also decreased plant cover, which in turn lowered fire frequency. Hence, the results herein did not depend on any assumption connecting fire frequency and aridity: even without increasing the flammability, open shrublands would replace the oak forests if increased aridity significantly affected the reproductive and resprouting performance of oaks (Fig. 3; Notes S2).

Aridity‐driven decreases in post‐fire responses may drive transitions in Mediterranean vegetation even more abruptly and irreversibly than forecasted previously. This model finding is potentially general and may be relevant at broader spatial scales and/or in other fire ecosystems, as fire‐driven vegetation feedbacks have been reported across the world (Dantas *et al.*, 2016; Tepley *et al.*, 2018; Abis & Brovkin, 2019). Dynamic Global Vegetation Models (DGVMs) are commonly used for predictions of vegetation under climate change, and predict unprecedented northward biome shifts in the Mediterranean Basin (Guiot & Cramer, 2016). However, DGVMs generally do not appropriately include plant fire‐response traits, especially overlooking tree resprouting capabilities (Kelley *et al.*, 2014; Baudena *et al.*, 2015; Hantson *et al.*, 2016). The present study highlights the necessity and urgency of including fire‐related functional types and post‐fire responses for prediction of fire ecosystems under future scenarios.

## Author contributions

MB, MR, AGM, SB and VRV conceived the project; SB critically contributed to the development of the research questions; MJB and VMS provided the field data, helped with the calibration and parameterization, and provided detailed knowledge about the plant communities studied, together with SB, AGM, VRV, A Vasques and A Valdecantos; MB developed the model, with contributions from FR, LH, MBE and MR; FR performed the analytical calculations in Notes S2; MB wrote the first draft and led the writing process. All authors contributed critically to the draft and gave final approval for publication.

## Supporting information

Please note: Wiley Blackwell are not responsible for the content or functionality of any Supporting Information supplied by the authors. Any queries (other than missing material) should be directed to the *New Phytologist* Central Office.

**Notes S1** Supplementary materials and methods.
**Notes S2** Analytical calculations of the oak cover values with fires.
**Notes S3** Monte Carlo simulations varying initial plant cover.
**Notes S4** Exploring different colonization rates.
**Notes S5** Short‐term experiments.
