# Supplementary material for: Increased aridity drives post‐fire recovery of Mediterranean forests towards open shrublands
Source: New Phytol. 2019 Nov 22;225(4):1500–15. doi: 10.1111/nph.16252 (PMC7004039; doi:10.1111/nph.16252)
Supplement: Supplementary file 1 — Notes S1 Supplementary materials and methods. Notes S2 Analytical calculations of the oak cover values with fires. Notes S3 Monte Carlo simulations varying initial plant cover. Notes S4 Exploring different colonization rates. Notes S5 Short‐term experiments. [file NPH-225-1500-s001.pdf]

Supplementary Information

For the paper "Increased aridity drives post-fire recovery of Mediterranean forests towards open shrublands" by Baudena et al. 2019, New Phytologist (accepted on: October 3rd, 2019).

TABLE OF CONTENT

NOTE S1 **SUPPLEMENTARY MATERIAL AND METHODS** **2**

S1.1 **OAK FUNCTIONAL TYPE** **2**
S1.2 **ADDITIONAL MODEL DESCRIPTION** **2**
SEED BANK AND SEEDER RESPONSE AFTER FIRE 2
S1.3 **OBSERVATIONAL DATA** **5**
S1.4 **MODEL CALIBRATION** **6**
OLD-FIELD-CAL 6
FIRE-CAL 8
S1.5 **ANALYSES: STANDARD SET OF INITIAL CONDITIONS** **9**

NOTE S2 **ANALYTICAL CALCULATIONS OF THE OAK COVER WITH FIRES** **10**

S2.1 **ASYMPTOTIC OAK COVER FOR PERIODIC FIRES** **10**
S2.2 **LOWER BOUNDARY OF OAK COVER FOR STOCHASTIC FIRES** **12**

NOTE S3 **MONTE CARLO SIMULATIONS VARYING INITIAL PLANT COVER** **15**

S3.1 **METHOD** **15**
S3.2 **DOES THE TIME TO OAK DOMINANCE DEPEND ON INITIAL CONDITIONS?** **15**
S3.3 **DO PLANT COVER INITIAL CONDITIONS INFLUENCE THE FINAL STATE(S)?** **18**

NOTE S4 **EXPLORING DIFFERENT COLONIZATION RATES** **21**

S4.1 **METHOD** **21**
S4.2 **RESULTS** **22**

NOTE S5 **SHORT-TERM EXPERIMENTS** **26**

S5.1 **DETAILS OF SHORT-TERM EXPERIMENTS** **26**
S5.2 **ARIDITY LEVEL HARSHENING OVER TIME** **29**

REFERENCES **30**

Note S1 Supplementary Material and methods

S1.1 Oak functional type

We considered one plant type for evergreen oaks, including the species Holm oak *Quercus ilex* and Kermes oak *Quercus coccifera.* These two closely related species can be considered as part of the same functional group considering a large set of traits (Paula *et al.*, 2009) that are relevant for our model. They are particularly similar if compared with the other plant types and species modelled. *Q. coccifera* is a tall late-successional shrub (or, more rarely, a small tree reaching up to 6 m) that can be considered functionally similar to *Q. ilex* regarding for example: rooting depth (Rambal, 1984; Canadell *et al.*, 1996), response to water stress (Martínez-Ferri *et al.*, 2000; Baquedano & Castillo, 2007), resprouting ability after fire and other disturbances (Tavşanoğlu & Pausas, 2018), seed emergence, seed size and dispersal mode (Paula *et al.*, 2009; Tavşanoğlu & Pausas, 2018). The two oak species were considered as one functional group in our model for their similar responses to water stress, fire and colonization abilities.

S1.2 Additional model description

Seed bank and seeder response after fire

Expansion of seeders depended on seed bank production, persistence, germination after fire of the seeders, and the seedling establishment, and was incorporated in the model in the last terms on the r.h.s. of equation (1) in the main text for i=2-5, while the term was set to zero for the resprouters ($\alpha_1= \alpha_6=0$).

As eqn (2) in the main text illustrates, the seeder parameters α_i (i=2-5) were defined as proportional to the seed bank $S_i(t)$ cumulated before the latest fire (which occurred at time t_f). Here we explain in detail how we estimated the parameters γ_i from the germination abilities, $g_{p,i}$ and the seedling survival after germination, $s_{g,i}$, both expressed as fractions:

$$\alpha_i(t) = C\frac{\gamma_i S_i(t)}{\sum_{i=2}^{5}\gamma_i S_i(t)} = C\frac{g_{p,i} s_{g,i} e_i S_i(t)}{\sum_{i=2}^{5} g_{p,i} s_{g,i} e_i S_i(t)} ;$$

where C was a conversion parameter determining the dimension of the α_i (y^{-1}), and S was unitless. This expression also includes a proportionality factor e_i, representing the inverse of the number of seeds that under optimal conditions would allow plant type i to colonize a unit of space in a certain unit of time. The parameters e_i were estimated by expert knowledge as the number of seedlings that would lead to dominance of a plant type per unit area, and then expressed in proportion to pines' e (e_2=1). The numerator thus estimates the relative fitness of the plant types to occupy bare soil after fire. As the species are competing for the space available after fire, we needed to correct for the fact that part of the potential expansion areas for species i would become occupied by species arriving earlier. This was corrected for by introducing in the denominator the sum of the products of $g_{p,i}$, $s_{g,i}$, e_i, and S_i of the four seeders. See Tab. S1 for parameter values.

The seed banks S_i varied in time as a consequence of antecedent plant dynamics. To keep the model to a certain level of simplicity, as the α_i terms were in fact relevant for the dynamics

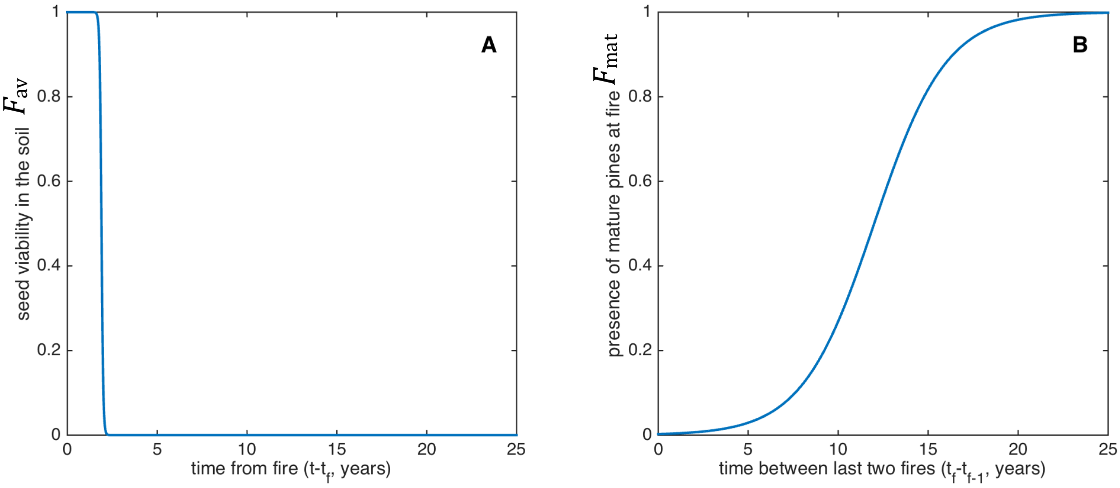

Fig. S 1 Pine seed bank functional forms. A) Pine seed viability in the soil F_{av} as a function of time since the latest fire (t-t_f). Pine seeds are viable only for p_{seed} =2 y after a fire in the soil: F_{av} was 1 for the first two years after a fire and then it became equal to zero, with a continuous fast transition between the two values. B) Presence of mature pines at time of last fire F_{mat}, determining the seed bank within the pine canopy. F_{mat} grows with time between the latest and the penultimate fires (t_f-t_{f-1}), with pines reaching maturity age on average at p_{mat} =12 y. See text below for the equations of the curves in the graphs.

directly after a fire when a lot of space was left empty, we did not define a differential equation for each seed bank, but we calculated it as a function of the average cover between the latest and the penultimate fires. Details differ for pines and shrubs, as we illustrate here below.

Pine seed bank

Pines (i=2) have an aerial seed bank that falls from the canopies to the ground at each fire, and the seeds are then viable for approximately two years. The seed bank also depends on the age of the pines before the latest fire, because pines produce seeds only at mature age (around 10-12 years). For pines the seed bank S_2 at time t was defined as:

$$S_2(t) = S_2(t_f)\cdot F_{av}(t-t_f)\cdot F_{mat}(t_f - t_{f-1}) = s_{p,2}\cdot \bar{b}_{f,2}\cdot \max(0,(t_f - t_{f-1} - p_{mat}))\cdot F_{av}(t-t_f)\cdot F_{mat}(t_f - t_{f-1})$$

Seed bank at time of the latest fire $S_2(t_f)$ was the result of the product of three factors: i) $s_{p,2}$, the yearly seed production, expressed for all seeders relative to the pine seed production (i.e. $s_{p,2}$=1 y^{-1}); ii) $\bar{b}_{f,2}$, the pine cover averaged between the latest and the penultimate fires, and iii) (t_f–t_{f-1}–p_{mat}), the time between the last two fires, minus the pine maturity age p_{mat} (Table S1), to take into account that seeds cumulate in the canopy from pine maturity until a fire occurs. If the value of t_f–t_{f-1}–p_{mat} was negative, the pines had not reached maturity at the time of the latest fire, and thus the maximum function set the seedbank to zero.

The term F_{av} represented pine seeds being viable in the soil only for two years after a fire (p_{seed} =2 yr; Fig. S1–A):

F_{av} = ½[1 – tanh((t–t_{f}–p_{seed}+a_1)/a_1)]

where time since the latest fire was (t-t_{f}). F_{av} was equal to 1 for the first two years after a fire and then it became equal to zero, with a continuous fast transition between the two values (Fig. S1).

Finally, F_{mat} was a function that determined whether there were mature pines at the time of the latest fire (Fig. S1 - B):

F_{mat} = ½ [1+tanh((t_{f}–$t_{\mathrm{f-1}}$–p_{mat})/a_2)],

which grows with time (t_f-t_{f-1}) between the latest and the penultimate fires, with pines reaching maturity age on average at p_{mat} =12 yr. See Table S1 for all parameters and their values.

Shrub seed bank

For the shrub seeders (i=3-5), the seed bank between fires decayed exponentially with time from the latest fire (t–t_f):

$$S_i(t) = S_i(t_f)\exp\left(-\frac{3(t - t_f)}{\mu_i}\right)$$

where μ_i is related to the seed durability in the soil (yr): the seed bank was reduced to 5% after μ_i years. If there was a fire, the seed bank term was recalculated:

$$S_i(t_f) = \left(S_i(t_f - 1) + s_{p,i}\cdot \bar{b}_{f,i}\cdot (t_f - t_{f-1})\right)s_{s,i}$$

where the seed bank right before the fire, $S_i(t_f$–1), was incremented by the seeds produced between the latest and penultimate fires. This was calculated as the product of three factors: the average plant cover between the fires $\bar{b}_{f,i}$, the seed production $s_{p,i}$ of the shrub i per unit of time (expressed with respect to pines), and the time between the latest and penultimate fires (t_f–t_{f-1}). Finally, as only a fraction of the seeds survived the fire, both terms were multiplied by the seed survival rate of each plant type, $s_{s,i}$.

Tab. S 1 List of parameters used to calculate the colonization of seeders after fire (α_i): symbols, names, values, units and their source. Q: *Quercus* spp; P: *P. halepensis*; R: *R. officinalis*; U: *U. parviflorus*; C: *Cistus* spp; B: *B. retusum*

Symbol	Name	Values in use for						Unit	Source
		Q (i=1)	P (i=2)	R (i=3)	U (i=4)	C (i=5)	B (i=6)		
p_{mat}	Pine maturity age	-	12	-	-	-	-	yr	*a*
p_{seed}	Pine seed viability	-	2	-	-	-	-	yr	*a*
a_1	Width of the hyperbolic tangent in F_{av}		0.1					yr	-
a_2	Width of the hyperbolic tangent in F_{mat}		4					yr	-
$s_{p,i}$	Seed production (in prop. to pine)	-	1	48	18	242	-	yr^{-1}	*b*
$s_{s,i}$	Fraction of seeds surviving after fire	-	1	0.37	0.86	0.86	-	-	*c*
$g_{p,i}$	Germination	-	0.13	0.09	0.08	0.03	-	-	*d*
$s_{g,i}$	Seedling survival after germination	-	0.31	0.36	0.36	0.26	-	-	*e*
e_i	Seeds to plant dominance (per unit area, in prop. to pine)	-	1	0.5	0.1	0.1	-	-	*f*
μ_i	Seed viability in the soil	-		10	100	50	-	yr	*f*
C	Conversion parameter	-	0.014				-	yr^{-1}	*g*

a: (Pausas, 1999; Climent *et al.*, 2008)
b: P: (Daskalakou & Thanos, 1996; Tapias *et al.*, 2001; Verkaik & Espelta, 2006); R, U, C: (Moya-Delgado, 2017)
c: P: expert estimation; R, U, C: (Santana *et al.*, 2013a; Moya-Delgado, 2017);
d: P: (Daskalakou & Thanos, 1996); R,U,C: (Santana *et al.*, 2012, 2014)
e: P: (Martínez-Sánchez *et al.*, 1999; Pausas *et al.*, 2003); R,U,C: (Santana *et al.*, 2012, 2014)
f: expert estimation.
g: Estimated from fire data calibration (Fire-cal)

S1.3 Observational data

Old-field data

Vegetation in the 73 old-field sites was mostly composed of the six plant types described in the main text. The data reported absolute cover, and only about 16% of the data had plant cover of the six plant types summing up to >100%, indicating that plant layers were superimposed only in a minority of the cases. To be comparable with the model, we re-normalized these data to fractions of plant cover.

Tab. S2 Old-field data: geographical location, altitude, mean annual temperature and rainfall, aridity index, bedrock type and time since abandonment of the study sites. Plot sizes were of the order of 100-1000 m[2].

# of sampling sites	Location	Altitude (m a.s.l.)	Mean annual temperature (° C)	Mean annual rainfall (mm yr^{-1})	Aridity index *	Bedrock	Time since abandonment (yr)	Reference
18	38° 43´N 0° 46´W	600	14.5	554	0.74	Marl	5, 7, 10, 18, 20, 25, 45	(Padilla, 1997)
27	38°41´N 0° 7´W	463	17	523	0.62	Limestone	5, 15, 25, 50, 75	(Peña, 2007)
4	38°49´N 0°14´W	560	16	814	0.96	Limestone	1, 5, 10, 20	(Rodriguez-Aizpeolea *et al.*, 1991)
6	38° 40´N 0° 39´W	940	14.6	480	0.64	Marl	50, 100	(Santana *et al.*, 2010)
18	43°22´N 5° 37´E	380	14	544	0.70	Limestone	10, 40, 70	(Tatoni, 1992)

* Defined as the ratio between mean annual precipitation and potential evapotranspiration (UNEP, 1992); retrieved from:
- Tatoni 1992: from Zomer *et al.*, 2008 https://cgiarcsi.community/data/global-aridity-and-pet-database/
- All the other sites: PET retrieved from the Spanish Ministry of Agriculture GIS database (https://sig.mapama.gob.es/sigla/).

Fire data

We used data from the sites of Onil, Pardines, Ayora, and La Torre located in SE Spain, where a wildfire occurred, followed by one or two experimental fires (Santana *et al.*, 2013b). The size of the experimental fire plots was *ca.* 30 × 30 m. See Table S3 for the years in which fires occurred. The plots were regularly sampled: approximately every six months in the first two years after a fire, once a year for 5 years after a fire, and afterwards every 5 years. The time series of observed plant cover started at the time the second fire occurred, for all cases but the site of Ayora where it started between the second and the third fire. Within these time series, to include information also about the cover at the moment of fire, we used the values measured within each site in a neighbouring plot, which was not experimentally burned. This was justified as the neighbouring plots were located within short distance from each other (about 15-20 m apart) and had a very similar plant composition. As for the old-field data, we re-normalized the fire data, which was necessary for only about 19% of the data point whose plant layers were superimposed. See Fig. S 2 for the time series of plant covers in the four sites.

Tab. S3 Years of fire occurrence for the four sites in SE Spain (Santana *et al.*, 2013b)

	Year of fire occurrence			
**Fire # and type**	**Onil**	**Ayora**	**Pardines**	**La Torre**
1st (wildfire)	1984	1979	1984	1984
2nd (experimental fire)	1994	1996	1994	1994
3rd (experimental fire)	2006	2006	2006	-

S1.4 Model calibration

We performed model calibration to find the optimal parameters, using the residual sum of squares between the model trajectories and the data as a goodness-of-fit measure. More precisely, we minimized the mean square deviation between model and data with respect to the variance of the data, using the index H^2, that is zero for a perfect model, and it is one when the variance of the data and the mean square deviation are equal (Baudena *et al.*, 2012). The optimization was performed using the simulated annealing algorithm ("SA"; Kirkpatrick *et al.*, 1983), and in details: i) for the "Old-field-cal" (calibration of the competition model with the old-field data), the SIMPSA algorithm (a combination of the non-linear "smplex" and the SA algorithm (Cardoso *et al.*, 1996), as implemented by Donckels (2009) for Matlab; ii) for the "Cal fire" (calibration of the full model with the fire data), the SA implementation for Fortran by Goffe *et al.* (1994).

Old-field-cal

Here we provide: i) the intervals over which the parameters were calibrated (Table S4); ii) additional calibrations to obtain a realistic range of validity for the parameters, including a Monte Carlo procedure.

Ranges used in old-field-cal

The calibration of the model with the old-field data was performed to optimize the values of the six colonization rates $c_{1\text{-}6}$. As the initial conditions of the plant cover $b_{i,0}$ were not known, they were left free and estimated with the calibration procedure too. Table S4 shows the ranges used for parameter optimization. The minimum for the colonization rates for each plant type coincided with their extinction rates m_i (Table 2).

Tab. S4 Ranges over which the parameters were calibrated in the Old-field-cal

	c_1	c_2	c_3	c_4	c_5	c_6	$b_{1,0}$	$b_{2,0}$	$b_{3,0}$	$b_{4,0}$	$b_{5,0}$	$b_{6,0}$
	yr^{-1}	yr^{-1}	yr^{-1}	yr^{-1}	yr^{-1}	yr^{-1}	-	-	-	-	-	-
min	0.0025	0.0080	0.020	0.040	0.067	0.025	10^{-6}	10^{-6}	10^{-6}	10^{-6}	10^{-6}	10^{-6}
max	1	10	20	20	20	20	0.3	0.3	0.3	0.3	0.3	0.3

Different types of calibrations for the Old-field-cal

Besides the calibration described in the main text (see Fig. 1, continuous line), we repeated the procedure in four different ways to obtain a range of validity for each parameter (Fig. 1, shaded areas). Namely: i) We optimized one plant type at a time, in hierarchical order from oak to grass (instead of optimizing all plant types simultaneously). ii) We divided the series in two parts according to the values of grass cover, namely one series included the sites with the upper quartile of the grass (*Brachypodium retusum)* cover values, and another series the rest of the data. We performed the optimization of the two series separately. iii) We optimized the model using only the first 5 plant types, excluding the grass (*Brachypodium retusum*), whose colonization rate c_6 had proven very difficult to calibrate, and it was better estimated with the

successive calibration including fire (Fire-cal). iv) We used a Monte Carlo procedure to repeat the model optimization a large number of times, as described in the following subsection.

Simulated Annealing – Monte Carlo

We repeated the model optimization with a Monte Carlo procedure, to include the intrinsic uncertainty in the old-field data regarding time since land abandonment and on plant cover. For each optimization, we used a synthetic dataset in which the data points were sampled around the observed values in the old-field data.

We considered the uncertainties regarding time since land abandonment and plant cover separately. We assumed that these two variables were normally distributed around their observed value, with a standard deviation equal to the square root of the data value. Distributions were truncated to guarantee no variable was picked outside of their range of existence (between 0 and 1 for plant cover, above zero for time). For plant cover, as some data points were equal or close to zero, a minimum value of 0.01 was chosen for the standard deviation to include some variability.

We generated 2x100 synthetic datasets, and the Old-field-cal procedure was then applied to each of them. We obtained thus 200 sets of calibrated colonization rates, see Table S5 for the ranges they spanned and Fig. 1 for a visual display of the effect of this variability on plant cover.

Tab. S 5 Ranges of the colonization rates as obtained by the SA-MC

	c_1 (Q)	c_2 (P)	c_3 (R)	c_4 (U)	c_5 (C)	c_6 (B)
	yr^{-1}	yr^{-1}	yr^{-1}	yr^{-1}	yr^{-1}	yr^{-1}
**Fit from SA-MC time of abandonment**						
min	0.040	0.045	0.047	0.076	0.14	0.090
max	0.053	0.064	0.060	0.092	0.16	0.12
best fit	0.053	0.055	0.057	0.085	0.16	0.12
**Fit from SA-MC plant cover**						
min	0.032	0.012	0.034	0.076	0.16	0.17
max	0.049	0.035	0.059	0.12	0.24	0.31
best fit	0.039	0.025	0.036	0.081	0.18	0.21

Fire-cal

We used the fire data to calibrate the values of the fire related parameters (α_i, r_i). We simulated fire occurrences at the same time as in the observations, and used the first observational data to initialize the model.
We considered the following parameters: C, $s_{p,3\text{-}5}$, r_6, $c_{1,3\text{-}6}$, m_6, g_p, e (see Table S6 for the ranges used). Besides the fire-related parameters, we also included in the calibration colonization and extinction rates, as the dynamics of the fastest colonizers could depend on these parameters as well. We performed separate and joint calibrations of these parameters, to identify which parameters would contribute the most to the model improvement, estimated with H^2 (Baudena *et al.*, 2012, see explanation above at the beginning of section S1.4). We selected the most parsimonious model identifying the lowest Akaike Information Criterion (AIC, Akaike, 1974). The parameter that showed the greatest model fit improvement (decrease in H^2) was C, followed by r_6 and c_6, with their joint model having a H^2=0.27 and lowest AIC (see Table S7).
We therefore determined the values for these three parameters with the fire-cal (see Table 2 for values), while we kept the values from literature, expert knowledge or obtained with the old-field-cal, for the parameters that were not improving the fit (Table S7). See in Fig. S2 the model trajectories with the fire data in the four sites.

Tab. S 6 Range used for the optimization of the parameters within the Fire-cal (see also Table S7).

	C	c_3	c_4	c_5	c_6	m_6	r_6	e_3	e_4	e_5
	yr^{-1}	yr^{-1}	yr^{-1}	yr^{-1}	yr^{-1}	yr^{-1}	-	-	-	-
min	0.001	0.01	0.01	0.01	0.01	0.005	0.3	0.0001	0.0001	0.0001
max	1	0.25	0.30	0.35	0.35	0.1	1	0.9999	0.9999	0.9999

Tab. S 7 Calibration of the model with the fire data series (Fig. S7). The model goodness-of-fit was evaluated using the index H^2 (where H^2=0 for a perfect model-data fit). The Akaike Information Criteria (AIC) was used for model selection (in bold). Each of the parameter values was varied in the range reported in Table S6.

**Param. used for optimisation**	Parameter values										**H^2**	**AIC**
	C	c_3	c_4	c_5	c_6	m_6	r_6	e_3	e_4	e_5		
Units	yr^{-1}	yr^{-1}	yr^{-1}	yr^{-1}	yr^{-1}	yr^{-1}	-	-	-	-		
C, $c_{3\text{-}6}$, m_6, r_6, $e_{3\text{-}5}$	0.021	0.02	0.30	0.09	0.21	0.0050	0.41	0.61	0.00	0.08	0.26	27.3
C, $c_{3\text{-}6}$, m_6, $e_{3\text{-}5}$	0.019	0.010	0.30	0.19	0.072	0.0057		0.94	0.0013	0.034	0.34	39.3
C, $c_{3\text{-}6}$, m_6, r_6	0.011	0.250	0.13	0.350	0.22	0.0050	0.40				0.26	21.9
C, c_6, m_6, r_6, $e_{3\text{-}5}$	0.022				0.21	0.0050	0.41	0.69	0.010	0.095	0.26	22.1
C, c_6, m_6, r_6	0.014				0.19	0.0050	0.41				0.27	17.6
**C, c_6, r_6**	**0.014**				**0.22**		**0.41**				**0.27**	**15.9**
C, r_6	0.009						0.70				0.36	40.6
C, c_6	0.015				0.08						0.35	26.8
c_6, r_6 (C=0.1)					0.35		0.45				0.47	40.6

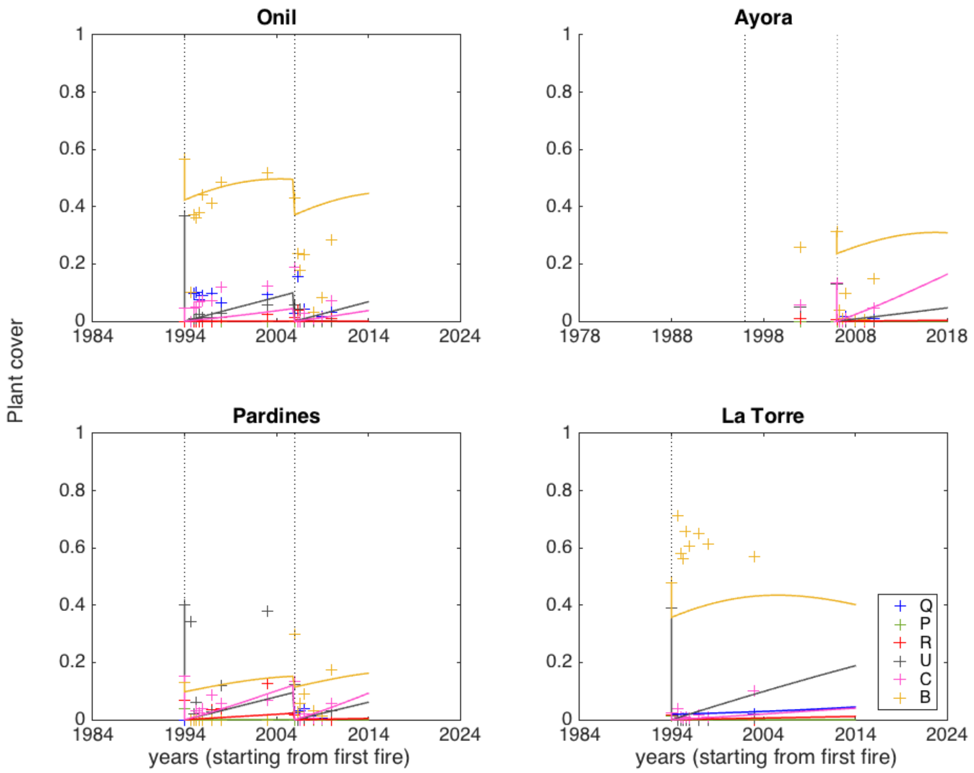

Fig. S 2 Fire data and calibrated model trajectories. Cover of the six plant types as a function of time (yr) from the first fire in the four sites in SE Spain (Santana et al 2013b; Tab S3). The first fires occurred in the first year represented (but note the different temporal range in Ayora). Dashed vertical lines represent subsequent fire years (Tab S3). Symbols represent observational data; lines represent modelled trajectories (obtained used the parameters from Table 2 and S1, where C, c_6 and r_6 optimized with the fire data shown here; H^2=0.27). See legend for colour codes.

S1.5 Analyses: standard set of initial conditions

Tab. S 8 Sets of initial conditions for the runs in Fig. 3. If the results varied for different initial conditions shown here, a much deeper and thorough analysis was performed (see Notes S3.3)

$b_{1,0}$ (Q)	$b_{2,0}$ (P)	$b_{3,0}$ (R)	$b_{4,0}$ (U)	$b_{5,0}$ (Q)	$b_{6,0}$ (B)
0.004	0.01	0.01	0.01	0.01	0.02
0.9	0.01	0.01	0.01	0.01	0.02
0.004	0.1	0.6	0.1	0.1	0.1
0.004	0.2	0.1	0.6	0.1	0.1
0.004	0.2	0.1	0.1	0.6	0.1
0.004	0.2	0.1	0.1	0.1	0.6

Note S2 Analytical calculations of the oak cover with fires

We here add analytical calculations to support our simulation results. To allow for analytical tractability, we considered fire frequency as externally imposed and not dependent on species composition. Firstly, we evaluated the effect of periodic recurrent fire, i.e. fires that occur in pulses but with a fixed fire return time. We could analytically calculate the oak cover asymptotic values before and right after a fire, as a function of the colonization and extinction rates, the resprouting capacity r_1, and the fire return time T_f. The calculations showed the same qualitative patterns reported for the stochastic fire simulations. Secondly, we retrieved the analytical expression of an asymptotic lower boundary of oak cover in case of stochastic fires.

First, let us change variables in the equation for oak cover (Eq.1 main text, with i=1):

$$\frac{\mathrm{d}b_1}{\mathrm{dt}} = c_1 b_1 (1-b_1) - m_1 b_1$$

to have a solution in exponential form. For simplicity of notation, we write

$$x = b_1, \quad a = c_1 - m_1, \quad \hat{x} = \frac{c_1 - m_1}{c_1},$$

As we assume the colonization rate to be larger than the extinction rate, $a > 0$, and thus there is a nontrivial equilibrium in the absence of fire. We make the following change of variable

$$z = x^{-1} - \hat{x}^{-1}.$$

Then, one has

$$\frac{\mathrm{d}z}{\mathrm{dt}} = -az,$$

so that

$$z = z_0\, \mathrm{e}^{-at}.$$

Incidentally, from here the known explicit solution (Tilman 1994) can be obtained:

$$b_1 = \frac{(c_1 - m_1) b_{1,0}\, \mathrm{e}^{(c_1 - m_1)t}}{c_1 - m_1 + c_1 b_{1,0} (\mathrm{e}^{(c_1 - m_1)t} - 1)}.$$

S2.1 Asymptotic oak cover for periodic fires

We here assume that fires occur in regular pulses, a simplification that allows for analytical tractability (as underlined by e.g. Tchuinté Tamen *et al.*, 2016, 2017; Yatat *et al.*, 2017). Let now z_n be the value of z before the nth fire happens, and z_n^+ its value after the fire (and, similarly, x_n and x_n^+ for the values of x).

Writing $k = T_f$ and $r = r_1$, and denoting $z_0^+ = z_0$ (the parameter a is still the net colonization rate) it is immediate that

$$z_n = z_{n-1}^+\, \mathrm{e}^{-ak}, \quad n \geq 1.$$

Since $x_n^+ = r x_n$, it follows that

$$z_n^+ = \frac{1}{r} z_n + \frac{1-r}{r}\hat{x}^{-1}.$$

Thus,

$$z_n^+ = \frac{1}{r} z_{n-1}^+ \,\mathrm{e}^{-ak} + \frac{1-r}{r}\hat{x}^{-1},$$

and, successively,

$$\begin{aligned}
z_n^+ &= \frac{1}{r}\left(\frac{1}{r} z_{n-2}^+ \,\mathrm{e}^{-ak} + \frac{1-r}{r}\hat{x}^{-1}\right)\mathrm{e}^{-ak} + \frac{1-r}{r}\hat{x}^{-1} = \frac{1}{r^2}\mathrm{e}^{-2ak} z_{n-2}^+ + \left(1+\frac{1}{r}\mathrm{e}^{-ak}\right)\frac{1-r}{r}\hat{x}^{-1} \\
&= \frac{1}{r^2}\mathrm{e}^{-2ak}\left(\frac{1}{r} z_{n-3}^+ \,\mathrm{e}^{-ak} + \frac{1-r}{r}\hat{x}^{-1}\right) + \left(1+\frac{1}{r}\mathrm{e}^{-ak}\right)\frac{1-r}{r}\hat{x}^{-1} = \frac{1}{r^3}\mathrm{e}^{-3ak} z_{n-3}^+ + \left(1+\frac{1}{r}\mathrm{e}^{-ak}+\frac{1}{r^2}\mathrm{e}^{-2ak}\right)\frac{1-r}{r}\hat{x}^{-1} \\
&= \cdots \\
&= \frac{1}{r^n}\mathrm{e}^{-nak} z_0^+ + \left(1+\frac{1}{r}\mathrm{e}^{-ak}+\frac{1}{r^2}\mathrm{e}^{-2ak}+\ldots+\frac{1}{r^{n-1}}\mathrm{e}^{-(n-1)ak}\right)\frac{1-r}{r}\hat{x}^{-1} \\
&= \frac{1}{r^n}\mathrm{e}^{-nak} z_0 + \frac{1-r^{-n}\,\mathrm{e}^{-nak}}{1-r^{-1}\,\mathrm{e}^{-ak}}\frac{1-r}{r}\hat{x}^{-1} \\
&= \mathrm{e}^{-n(\ln r + ak)} z_0 + \frac{1-\mathrm{e}^{-n(\ln r+ak)}}{1-\mathrm{e}^{-(\ln r+ak)}}\frac{1-r}{r}\hat{x}^{-1}.
\end{aligned}$$

Hence, the limiting behavior of *z* (and consequently of oak cover *x*) depends on the sign of the quantity $\ln r + ak$: if this is negative, then $z \to \infty$, and $x \to 0$, whereas if it is positive then there is a finite positive limit. In this case, naming z_∞^+ and x_∞^+ the asymptotic equilibrium values after a fire, one gets

$$z_\infty^+ = \frac{1}{1-\mathrm{e}^{-(\ln r+ak)}}\frac{1-r}{r}\hat{x}^{-1},$$

so that

$$x_\infty^+ = \left(1-\frac{1-r}{1-\mathrm{e}^{-ak}}\right)\hat{x} = \left(1-\frac{1-r}{1-\mathrm{e}^{-ak}}\right)\left(1-\frac{m_1}{c_1}\right).$$

We thus obtain an expression for the oak equilibrium cover reached asymptotically right after a fire, depending on r_1 and T_f, besides the colonization and extinction rates c_1 and m_1. Given the substitutions above, we can finally rewrite it as:

$$b_{1,\infty}^{+}=\left(1-\frac{m_1}{c_1}\right)\left(1-\frac{1-r_1}{1-\mathrm{e}^{-T_f(c_1-m_1)}}\right)$$ asymptotic oak cover after a fire

$$b_{1,\infty}=\frac{1}{r_1}\left(1-\frac{m_1}{c_1}\right)\left(1-\frac{1-r_1}{1-\mathrm{e}^{-T_f(c_1-m_1)}}\right)$$ asymptotic oak cover before a fire

for $T_f>-\ln(r_1)/(c_1-m_1)$, and zero otherwise.

The asymptotic solutions after/before fire for periodic fires show the same trend reported in the main text for the full model (Fig. 3), capturing correctly the range of the simulated results, and leading to oak disappearance when fires are very frequent and resprouting ability r_1 is low. See below Tab. S9 for oak cover values for c_1=0.047 (calculated at three r_1 valuesas in Fig. 3, and with T_f=10, 20, 30 and 40 yr). Differently from Fig. 3, this simplified model does not display bistability, as it does not include a feedback between vegetation and fire.

Tab. S 9 Values of oak cover b_1 obtained asymptotically after/before a fire for periodic fire, and their lower boundary approximations for stochastic fires. For c_1=0.047, and different values of r_1 and T_f.

	Periodic fire - T_f				***Stochastic fires - T_f***			
r_1	**40 yr**	**30 yr**	**20 yr**	**10 yr**	**40 yr**	**30 yr**	**20 yr**	**10 yr**
**0.60**	0.49/0.82	0.43/0.72	0.30/0.51	0	0.35/0.59	0.28/0.47	0.14/0.24	0
**0.75**	0.66/0.88	0.62/0.83	0.54/0.73	0.29/0.38	0.58/0.77	0.53/0.44	0.44/0.59	0.18/0.24
**0.90**	0.83/0.93	0.82/0.93	0.79/0.87	0.68/0.76	0.80/0.89	0.78/0.87	0.75/0.83	0.64/0.71

S2.2 Lower boundary of oak cover for stochastic fires

In this section we repeat the calculations above for stochastic and exponentially distributed fires.
For the stochastic case, we can write that fires occur at times T_j
$T_1=t_1,\ T_2=t_1+t_2,\ \ldots,\ T_n=t_1+t_2+\ldots t_n,\ \ldots,$
and thus the expression for z_n^+ takes the form

$$z_n^+=\frac{1}{r^n}\mathrm{e}^{-aT_n}z_0^+ +\left(1+\frac{1}{r}\mathrm{e}^{-aT_1}+\frac{1}{r^2}\mathrm{e}^{-aT_2}+\ldots+\frac{1}{r^{n-1}}\mathrm{e}^{-aT_{n-1}}\right)\frac{1-r}{r}\hat{x}^{-1}$$

We now consider that t_j are independent identically distributed, such that their expected value (i.e. the long-term average value) $E(t_j)=T_f$, the return time. Then, writing

$$\mu_{\exp}=E\left(\mathrm{e}^{-at_j}\right),\quad j=1,2,\ldots,$$

one gets

$$E\left(z_n^+\right) = \frac{1}{r^n} E\left(\prod_{j=1}^{n} \mathrm{e}^{-at_j}\right) z_0 + \left(1 + \sum_{l=1}^{n-1} \frac{1}{r^l} E\left(\prod_{j=1}^{l} \mathrm{e}^{-at_j}\right)\right) \frac{1-r}{r} \hat{x}^{-1}$$

$$= \left(\frac{\mu_{\exp}}{r}\right)^n z_0 + \sum_{l=0}^{n-1} \left(\frac{\mu_{\exp}}{r}\right)^l \frac{1-r}{r} \hat{x}^{-1}$$

$$= \left(\frac{\mu_{\exp}}{r}\right)^n z_0 + \frac{1-\left(\frac{\mu_{\exp}}{r}\right)^n}{1-\left(\frac{\mu_{\exp}}{r}\right)} \frac{1-r}{r} \hat{x}^{-1}.$$

In the deterministic periodic case, where $T_f = k$, one has $\mu_{\exp} = \mathrm{e}^{-ak}$,and the previous result is recovered.

Now, if $\mu_{\exp} < r$, the limiting value of the average is:

$$E\left(z_n^+\right) \to \frac{1}{1-\left(\frac{\mu_{\exp}}{r}\right)} \frac{1-r}{r} \hat{x}^{-1},$$

so that

$$E\left(z_\infty\right) = \frac{1-r}{r-\mu_{\exp}} \hat{x}^{-1}.$$

Finally, from Jensen's inequality (Jensen, 1906),

$$E\left(x_\infty\right) \geq \frac{1}{E\left(z_\infty + \hat{x}^{-1}\right)} = \frac{\hat{x}}{\frac{1-r}{r-\mu_{\exp}} + 1} = \frac{r-\mu_{\exp}}{1-\mu_{\exp}} \hat{x} = \left(1 - \frac{1-r}{1-\mu_{\exp}}\right)\left(1 - \frac{m_1}{c_1}\right).$$

Consider now that t_j are independent identically distributed, and their distribution is exponential such that $E(t_j) = T_f$. Then,

$$\mu_{\exp} = \int_0^\infty \mathrm{e}^{-at} \frac{1}{T_f} \mathrm{e}^{-t/T_f} \, dt = \frac{1}{T_f} \int_0^\infty \mathrm{e}^{-\left(a+1/T_f\right)t} dt = \frac{1}{1+aT_f}.$$

Therefore, if $T_f > \frac{1-r}{ar}$, then

$$E\left(x_\infty\right) \geq \left(\frac{r\left(1+aT_f\right)-1}{aT_f}\right)\left(1 - \frac{m_1}{c_1}\right).$$

This lower boundary solution can be thus rewritten in the notation of the main text (equation 1) to give a lower boundary for the asymptotic values before and after a fire:

$$b_{1,\infty}^{+} \geq \left(1-\frac{m_1}{c_1}\right)\left(r_1-\frac{1-r_1}{T_f(c_1-m_1)}\right) \text{ after a fire}$$

$$b_{1,\infty} \geq \left(1-\frac{m_1}{c_1}\right)\left(1-\frac{1-r_1}{T_f r_1(c_1-m_1)}\right) \text{ before a fire}$$

for $T_f > \frac{1-r_1}{(c_1-m_1)r_1}$ and $E\left(\mathrm{e}^{-(c_1-m_1)t_j}\right) < r_1$, and zero otherwise. See some example values in Table S9.

Note S3 Monte Carlo simulations varying initial plant cover

For the long-term simulations, we varied the initial conditions of plant cover with Monte Carlo simulations (MC-IC), to study their influence on: the time to oak dominance (§ S3.2) and the final state of the system when bistability is observed (§ S3.3).

S3.1 Method

We run a Monte Carlo ensemble of simulations of the model including the fire feedback by randomly varying the plant cover initial conditions (i.c.). The i.c. of each of the plant types $b_{1\text{-}6}$ were extracted from a homogeneous distribution between 0 and 1, under the condition that their sum was equal or smaller than 1. For each parameter combination, 4,010 MC-IC simulations were run. The simulations were run with the standard parameter set as in Table 2 and Tab. S1 unless mentioned otherwise.

S3.2 Does the time to oak dominance depend on initial conditions?

To check at which time the oaks became dominant and whether the timing depended on the initial community composition, under historical climatic conditions, we recorded for each run the time at which the oak cover would become larger than 0.5, 0.7 and 0.8 for the first time.

We then plotted these values as a function of the initial conditions of each plant type separately, $b_{i,0}$ (Fig. S 3), and of the sum of all plant covers except the oaks (Fig. S 4). The time of oak dominance varied between less than 50 y to more than 300 ry. These times (for all three thresholds considered) decreased exponentially with increasing values of oak cover i.c. (Fig. S 3-A).

This is explicable as the oak differential equation (*i*=1 in equation 1) in the competition model (without fires) does not depend on the cover of the other species (analogously to Tilman 1994). The solution of that equation is:

$$b_1 = \frac{(c_1 - m_1) b_{1,0}\, \mathrm{e}^{(c_1 - m_1)t}}{c_1 - m_1 + c_1 b_{1,0} (\mathrm{e}^{(c_1 - m_1)t} - 1)}. \qquad \text{Eq. A1}$$

where $b_{1,0}$ is the initial condition of b_1. We can now invert this formula to obtain a relationship between the initial condition $b_{1,0}$ and the time T at which a certain oak cover is obtained ($b_{1,T}$=0.5, 0.7 or 0.8):

$$b_{1,0} = \frac{-(c_1 - m_1) b_{1,T}}{[c_1 b_{1,T} - (c_1 - m_1)]\mathrm{e}^{(c_1 - m_1)T} - c_1 b_{1,T}}, \qquad \text{Eq. A2}$$

When plotting the time T at which oaks reached dominance as a function of b_0, this analytic relationship (continuous lines in Fig. S 3 A) overlapped with the simulated data obtained with the MC-IC runs that included fires. The fire frequency depended on plant cover and fires reduced the oak cover slightly, thus introducing some noise in the relationship between $b_{1,0}$ and the time needed to reach a certain cover $b_{1,T}$. However, this noise was not strong enough to override the memory of the deterministic trajectory.

When one plant type covered more than about 90% of the space, time to oak dominance increased with the initial cover of this specific plant type. However, this was the only connection between time to oak dominance and the initial cover of the other plant types, and

was probably due to the fact that initial oak cover would certainly be below 0.1 if one of the other plant types had such a high initial value. There was no connection with the initial conditions of the sum of the other plant types (Fig. S 4). This was also clearly illustrated by the plot of the return times attained for given values of initial conditions of oaks (Fig. S 5, for $b_{1,0}$=0.1; analogous plots were obtained for other values of $b_{1,0}$, not shown). The larger variability in oak return times for small $b_{2\text{-}6}$ (Fig. S 3 B-F) was probably due to the fact that if one plant type is low, more combinations of the other plant covers are possible.

When the same MC experiments were run for cases with harsher aridity conditions (different set of values for c_1, r_1 and flammabilities, as introduced in the main text), the time of oak dominance still followed the deterministic path, provided that the oak forest state could be reached given a certain aridity level (not shown).

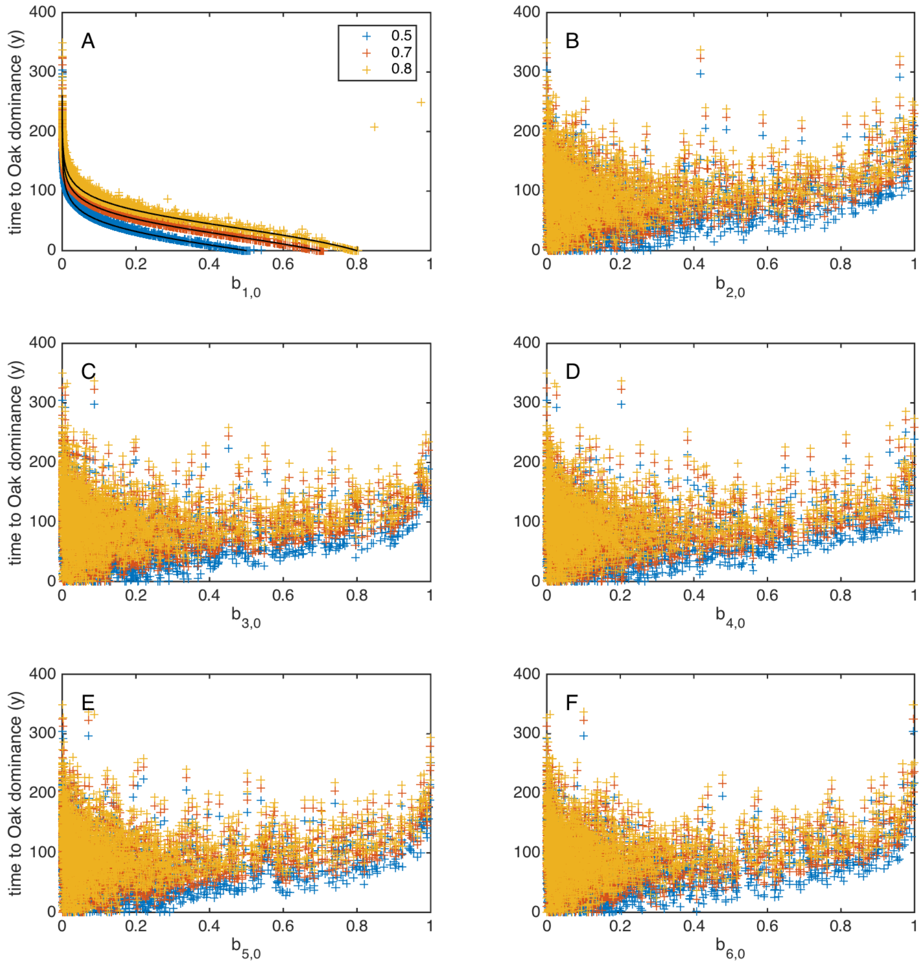

Fig. S 3 Time to oak dominance (for three different values of oak cover, i.e. 0.5, 0.7 and 0.8, see legend in panel A for colour code) as a function of the six initial plant covers ($b_{1\text{-}6}$). Continuous lines in panel A) represent the analytical expression as calculated from the competition model without fires (Eq. A2 above).

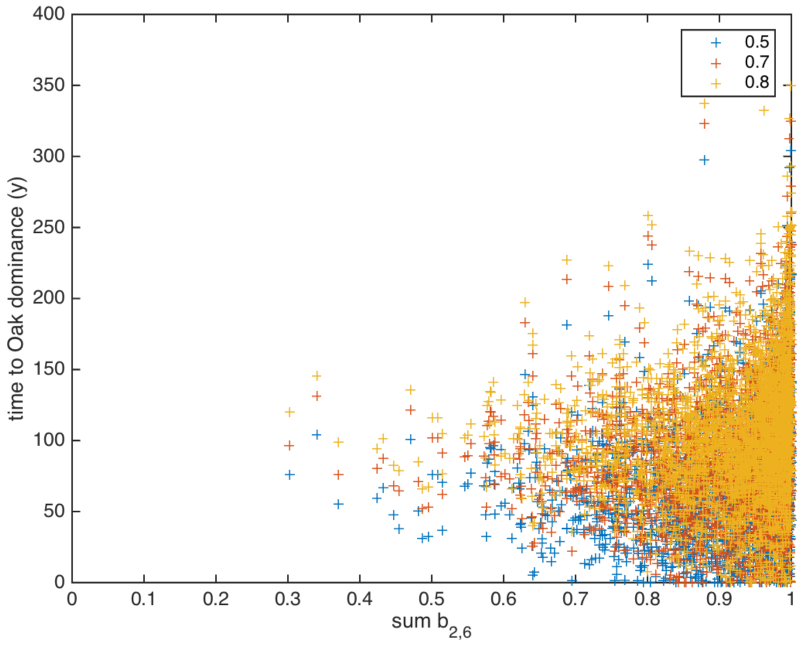

Fig. S 4 Time to oak dominance as a function of the sum of the initial plant covers, oak excluded ($b_{2\text{-}6}$).

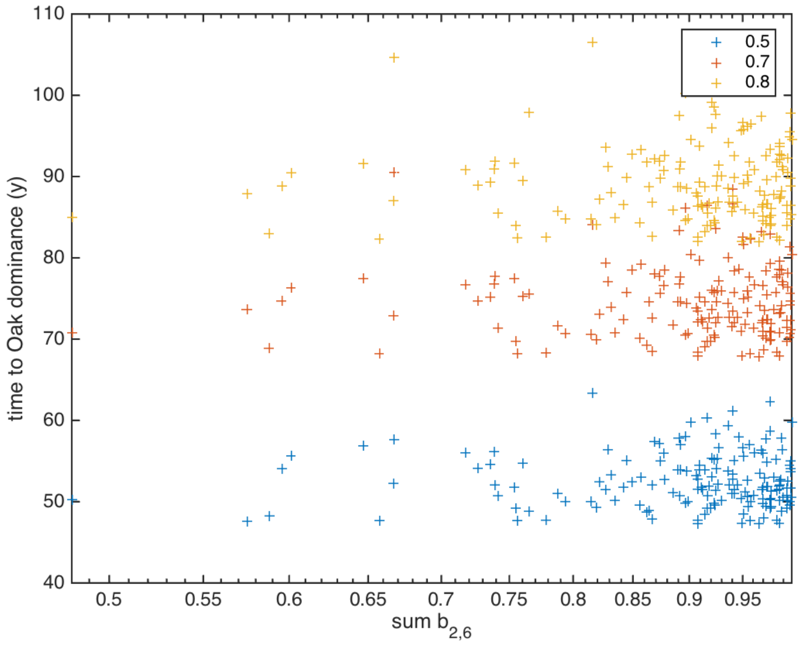

Fig. S 5 Time to oak dominance as a function of the sum of the initial plant covers, oak excluded ($b_{2\text{-}6}$), with run selected for oak initial cover ~0.1 ($0.09 \leq b_1 \leq 0.11$).

S3.3 Do plant cover initial conditions influence the final state(s)?

We *explored all the 48 parameter sets (Fig. 3) with the MC-IC approach, to assess the existence of possible alternative stable states with a larger number of runs, and to study the dependence of the final states on initial conditions. We report here the findings for the historical climate conditions and for the five parameter combinations for which the system displayed more than one possible stable state (Fig. 3).*

Historical climate conditions. For the standard parameter set (Fig 2 and Fig. 3I, left bar), the MC-IC simulations confirmed that the final state was unique: an oak forest, with oak cover about 0.9, and almost no other plant type present (not shown). This is very similar to the only equilibrium of the competition model without fire (which included only oaks, b_1=0.95).

Increased aridity. For the five parameter sets for which the system displayed alternative states in Fig. 3, we analyzed whether reaching one of the two states was influenced by the initial conditions. In most of the cases, the final states did not depend on initial conditions: In other words, we did not observe any major founder effect.

Here we illustrate the findings for 3 of the 5 cases identified as bistable in Fig. 3 (the other two cases are analogous and not shown). For illustration, we classified as oak forests those states where oak cover was larger than b_{thr} =0.25, and as open shrubland those states with less than b_{thr} oak cover and more than b_{thr} shrub seeders and/or grass cover. The results were analogous if different b_{thr} values were chosen for oak cover.

I. c_1=0.035 yr $^{-1}$, r_1=0.60, flammability increased 3 times (Fig. 3B)
The final state of the system was an oak forest or a shrubland independently of the initial conditions, with the following few exceptions: i) if the initial oak cover was larger than ~0.9, forest had ~100% chance to occur, and ii) if the initial grass or shrub cover was larger than ~0.9, shrubland had ~100% chance to occur (Fig. S 6).

Very similar results (not shown) were found for two other cases of bistability, with parameter values: c_1=0.023 yr $^{-1}$, r_1=0.60, flammability increased 1.5 times (Fig. 3C) and c_1=0.023 yr $^{-1}$, r_1=0.75, flammability increased 3 times (Fig. 3G).

II. c_1=0.023 yr $^{-1}$, r_1=0.60, flammability increased 1.2 times (Fig. 3C)
The oak forest was the only state possible for $b_{0,1} \gtrsim 0.3$, while below this value shrubland had a finite, though small, chance to emerge, which increased also with shrub and grass initial covers (Fig. S 7). The shrubland had ~100% chance to occur if the initial grass or shrub cover was higher than ~0.9 (not shown).

III. c_1=0.011 yr $^{-1}$, r_1=0.75, baseline flammability (Fig. 3H).
The system was most likely to develop into a shrubland, but an oak forest was also possible. There was no major dependence of the final state on initial conditions of plant cover. However, if the initial grass cover was larger than about 50% ($b_{0,6} \gtrsim 0.5$), it was not possible to reach a forest final state (Fig. S 8).

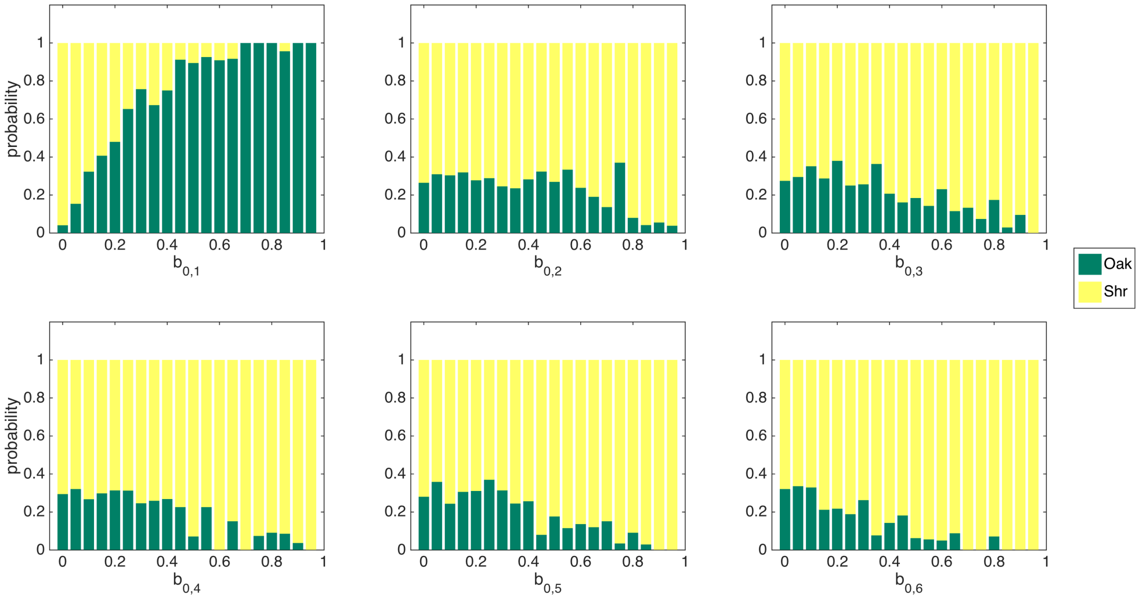

Fig. S 6 Probability of being in a forest (green) or a shrubland (yellow) state for different classes of values of initial cover of the plant types for case I with r_1=0.60, flammability 3 times above the baseline and c_1=0.035 yr^{-1} (Bistable case in Fig. 3B). Each panel represents the probability of the system to be in a final state of forest (green; more than b_{thr}= 25% oak cover) or shrubland (yellow; less than b_{thr}= 25% oak cover and more than 25% seeders and/or grass), as a function of the initial conditions of a plant type per panel ($b_{0,i}$, with i=1-6). The final state of the system was an oak forest or a shrubland almost independently of the i.c., a part from: i) forest having ~100% chance to occur if the initial oak cover was $b_{1,0} \gtrsim 0.9$ (top-left panel), and ii) shrubland having ~100% chance to occur if the initial grass or shrub cover was higher than ~0.9 (top-right and bottom panels)

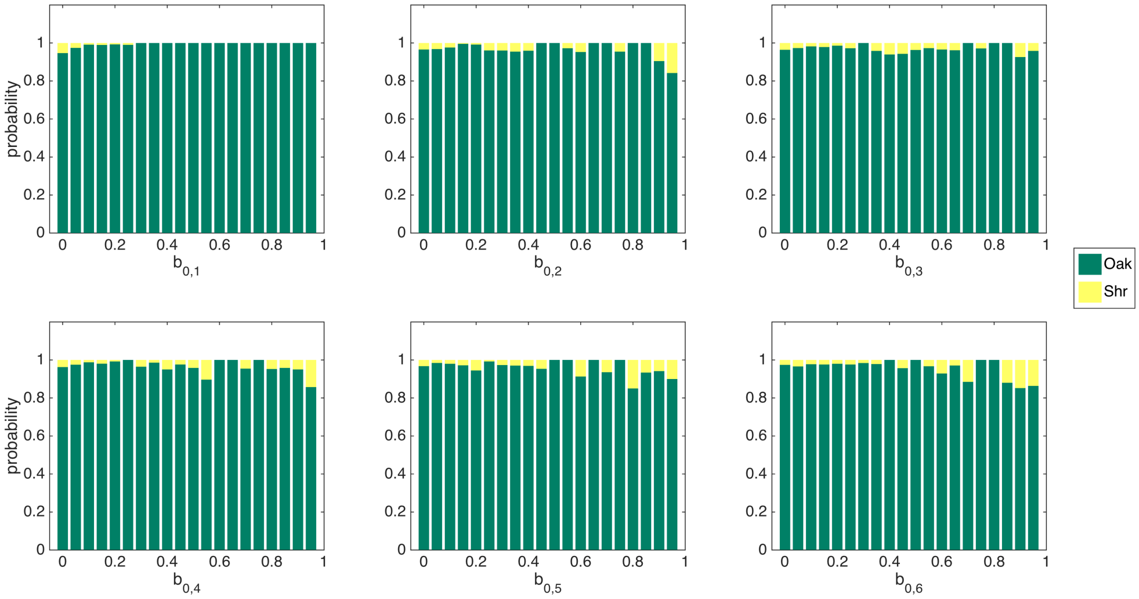

Fig. S 7 Probability of being in a forest (green) or a shrubland (yellow) state for different values of initial cover of the plant types for case II r_1=0.60, flammability 1.2 times above the baseline and c_1=0.023 yr^{-1} (Bistable case in Fig. 3C). Each panel represents the probability of the system to be in a final state of forest (green; more than b_{thr}= 25% oak cover) or shrubland (yellow; less than b_{thr}= 25% oak cover and more than 25% seeders and/or grass)), as a function of the initial conditions of a plant type per panel ($b_{0,i}$, with i=1-6). The final state of the system was always an oak forest for $b_{0,1} \gtrsim 0.3$, while below this value shrubland had a finite, though small, chance to emerge, which increased with shrub and grass initial covers.

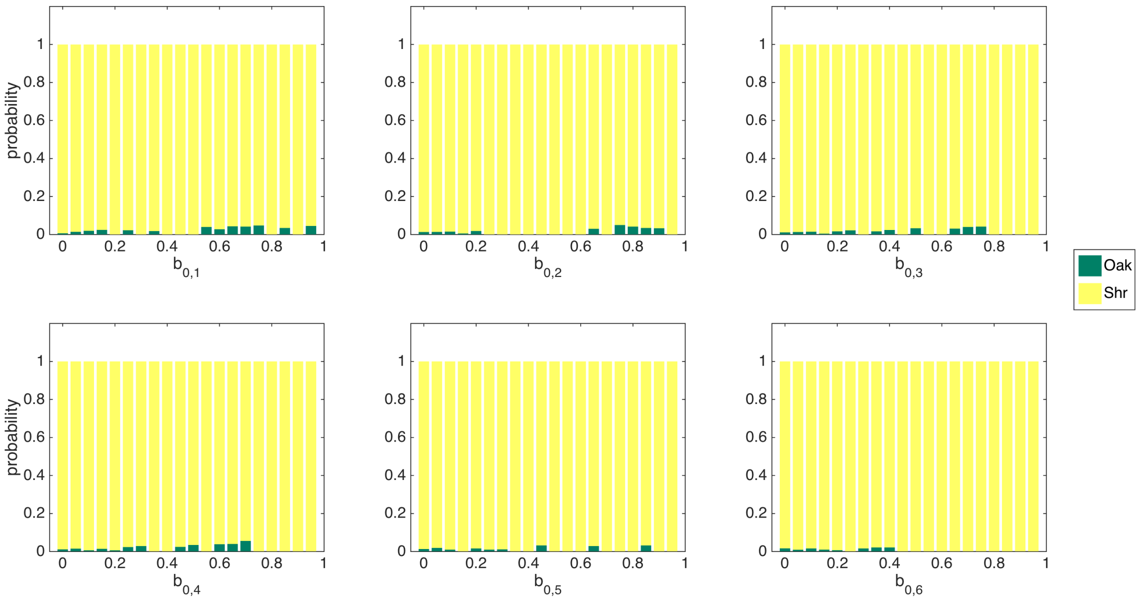

Fig. S 8 Probability of being in a forest (green) or a shrubland (yellow) state for different classes of values of initial cover of the plant types for case III with r_1=0.75, flammability x1 and c_1=0.011 yr^{-1} (bistable case in Fig. 3H). Each panel represents the probability of the system to be in a final state of forest (green; more than b_{thr}= 25% oak cover) or shrubland (yellow; less than b_{thr}= 25% oak cover and more than 25% seeders and/or grass). The states are represented as a function of the initial conditions of two different plant types ($b_{0,i}$, with i=1-6). The shrubland was the most likely state, but an oak forest was also possible. The only clear dependence on initial conditions observed is the fact that if grass cover was initially larger than ~50%, a forest could never develop (bottom right panel).

Note S4 Exploring different colonization rates

In the following, we present the results of a thorough exploration of the parameter space of colonization rates for the different plant types ($c_{1\text{-}6}$) with long-term simulations. These simulations had three aims:

- i) *including aridity effects on all plant types;*
- ii) *check the general validity of the results beyond the standard parameter set* (Tables 2 and S1);
- iii) *including parts of the parameter space were pines and oaks could theoretically coexist in the absence of fire.*

S4.1 Method

Aim i): Specific simulations

To study the effect of aridity reducing the colonization rates of the seeders and grasses (aim i), we performed simulations with the 48 aridity scenarios (described in the main text and as in Fig. 3), with the additional reduction of the colonization rates of the other plants. Specifically, given that in Fig. 3 c_1 was reduced with steps of 25% of its maximum value (Table 2), we reduced the other colonization rates $c_{2\text{-}6}$ with steps of 15% of their maximum values (Table 2), since aridity is expected to affect oaks more than the other plant types (e.g. Pausas *et al.*, 2016).

Aim i)-iii): Monte Carlo simulations on colonization rate

Furthermore, for the three aims, we performed a large set of Monte Carlo simulations (MC-COL) to vary the colonization rates $c_{1\text{-}6}$. We randomly sampled $c_{1\text{-}6}$ from uniform distributions between the values given in Table S10 below. These wide ranges for the parameter values were determined from the MC-SA old-field optimization procedure (see Table S5, Notes S1). We extended the range of c_1 further towards lower values, to be able to include a part of the parameter space where coexistence of oaks and pines was possible in the competition model without fires (see aim iii). Such minimum c_1 value could be calculated analytically (Tilman, 1994); see Fig. S 9).

We generated 1250 sets of values for $c_{1\text{-}6}$; for each of these sets we ran simulations for 8 different initial conditions (Table S11) and for 12 parameter sets given by the 3 values of the resprouting ability of the oak r_1 and 4 flammability values (as in Fig. 3, main text), for a total of 180,000 simulations.

Tab. S 10 Range of variation of the colonization rates $c_{1\text{-}6}$ for the MC-COL simulations

	c_1	c_2	c_3	c_4	c_5	c_6
**min** (yr^{-1})	0.005	0.01	0.021	0.04	0.05	0.067
**max** (yr^{-1})	0.07	0.17	0.15	0.19	0.24	0.40

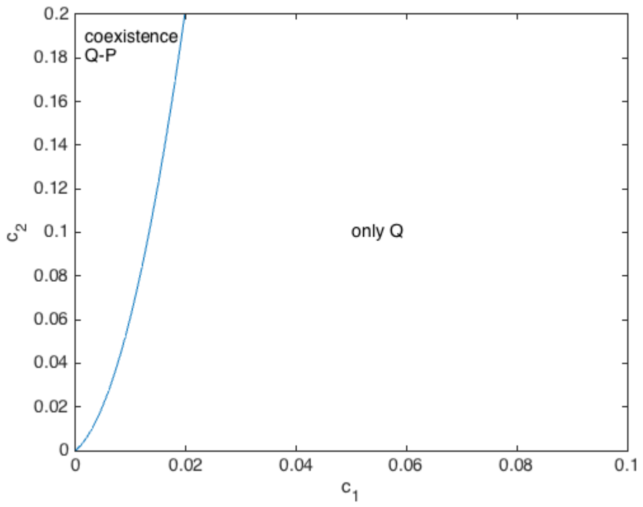

Fig. S 9 Parameter plane of c_1 and c_2 representing the area of coexistence of oaks and pines and the area with only oaks in the model without fires (coinciding with the model of Tilman, 1994).

Tab. S 11 Sets of initial conditions for the MC-COL simulations

Initial condition sets	$b_{1,0}$	$b_{2,0}$	$b_{3,0}$	$b_{4,0}$	$b_{5,0}$	$b_{6,0}$
1	0.001	0.01	0.01	0.01	0.01	0.01
2	0.89	0.01	0.01	0.01	0.01	0.02
3	0.01	0.8	0.01	0.01	0.01	0.01
4	0.01	0.01	0.8	0.01	0.01	0.01
5	0.01	0.01	0.01	0.8	0.01	0.01
6	0.01	0.01	0.01	0.01	0.8	0.01
7	0.01	0.01	0.01	0.01	0.01	0.8
8	0.15	0.15	0.15	0.15	0.15	0.15

S4.2 Results

Aim i): Specific simulations

When reducing the colonization rates of all species, the results were qualitatively similar to the case shown in the main text Fig. 3, see below Fig. S10: Under higher aridity levels shrublands dominated instead of forests, and bistability of the two states was observed. Additionally, we obtained analogous results by reducing the ability of seeders to regrow after a fire (α_i of the seeders reduced of 15% their maximum value, not shown)

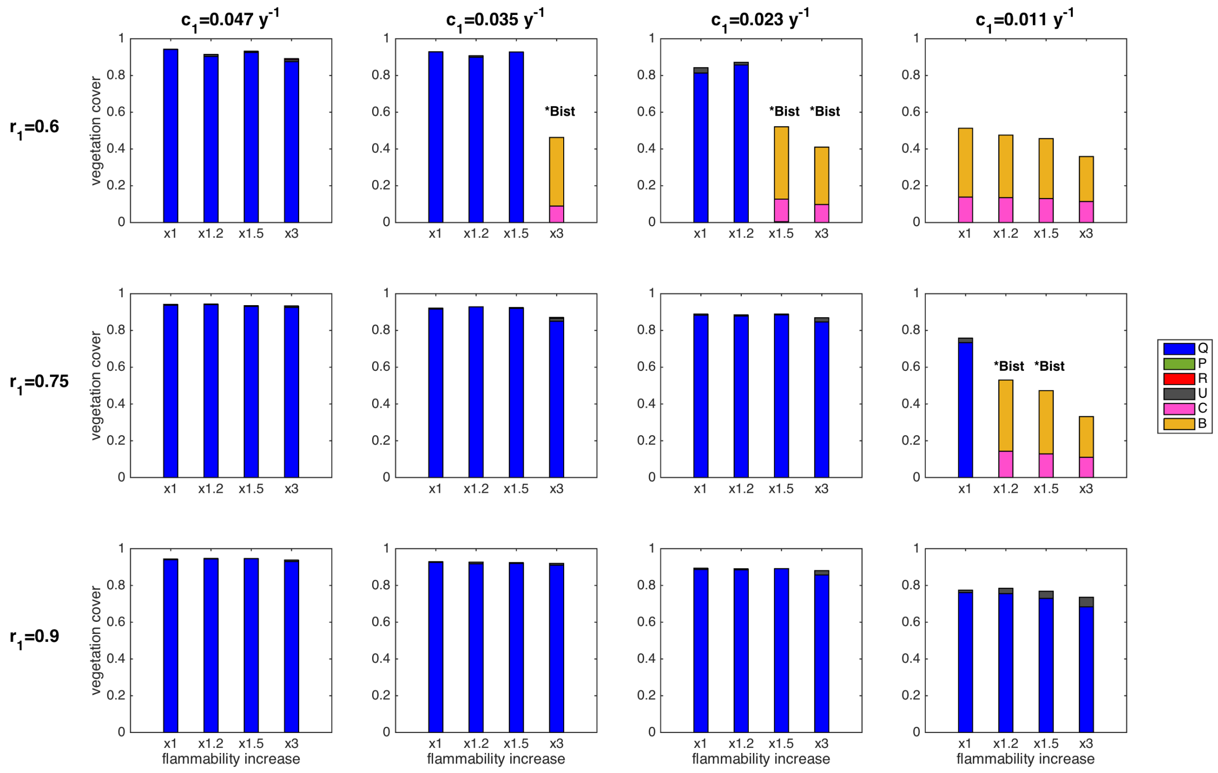

Fig. S 10 Plant community composition under the 48 aridity scenarios in the long-term experiments, decreasing colonization rates of all plant types. Bars represent average plant cover (calculated between 8,000 and 10,000 years from start of run) of each of the plant types for different values of: i) *x*-axis: fire return time (y); ii) from left to right, decreasing values of colonization rates $c_{1\text{-}6}$, as indicated in the figure for c_1, and reducing $c_{2\text{-}6}$ of 15% of their maximum value at each step iii) from top to bottom, increasing values of oak resprouting ability, r_1. The top-right panels, and the rightmost bars, represent the harshest aridity conditions. Few combinations of parameters are indicated as bistable ("*Bist"), since the same parameter setting can lead also to a forest with mostly oaks (bars are not shown).

Aim i)-iii): Monte Carlo simulations on colonization rate

(i) The colonization parameter that mostly determines the final state was the colonization rate of the oaks, c_1. The modelled stable states changed from forest, to bistability of forest and open shrubland, to open shrubland, as c_1 decreased. No such relationship was observed for any other colonization parameter (see first row of panels in Fig. S11 for r_1=0.75 and baseline flammability and Fig. S12 for r_1=0.60 and flammability three times above baseline; other cases not shown). The only other parameter that was sometimes relevant was the colonization rate of the grass c_6, as observed in the scenarios with larger flammability (see e.g. top-right panel in Fig. S 12), where open shrubland was observed in a wider part of the parameter space as c_6 increased. However, the decrease of c_1 was more important for the model outcome: decreases in c_6 needed to be larger (in proportion to their values) to shift the final state of the system. In other words, the model displayed smaller sensitivity to changes in c_6. Thus, as aridity is expected to affect oaks more drastically than grasses (Pausas *et al.*, 2016), we hereby proved that our results from Fig. 3 would be the same if all plant types were affected by aridity at most in the same proportion.

(ii) Given the large number of runs showing consistent results, we also proved that our results are robust and valid for a wide set of conditions and are not limited to a specific set of parameters.

(iii) In a few runs, pines could survive, either with oaks or within shrublands, given a sufficiently large pine colonization rate c_2, placing pines in the parts of the c_1-c_2 parameter space where pines and oaks could survive in absence of fires in the old-field model (Fig. S9). This was observed only if flammability was not increased (or not much, compare Fig. S 11-Fig. S 12), restating the importance of the demographic bottleneck for pines with recurrent fires.

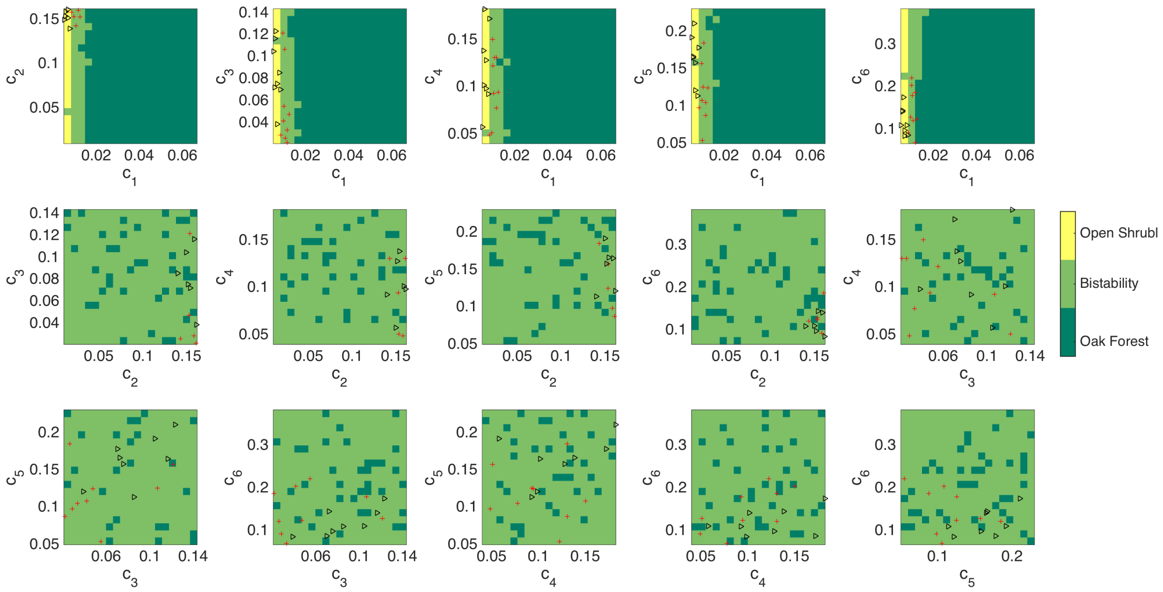

Fig. S 11 Possible states achieved by the system in different areas of the parameter space of the colonization rates. Each panel represents the final state of the system within three categories: "Oak Forest" (more than b_{thr}= 25% oak cover), "Open shrubl" (less than 25% oak cover and more than 25% seeders and/or grass), and "Bistability" (for those cells where both final states are possible). The states are represented as a function of two different colonization rates in each panel (between c_1 and c_6; in yr^{-1}). Symbols identify outcomes of specific runs where the final pine cover was larger than 10% in conjunction with: oaks (more than 0.25, red crosses); other plant types (oak less than 0.25, black triangles). This figure is related to panels E to H in Fig. 3, in which bistability was observed (r_1=0.75, with baseline flammability). The bimodal area clearly appears within a certain range of oak colonization rate (around 0.01 y^{-1}), while for even lower c_1 values only shrublands can persist. This figure is qualitatively unchanged if different values of b_{thr} are used (e.g. b_{thr}= 50%, etc).

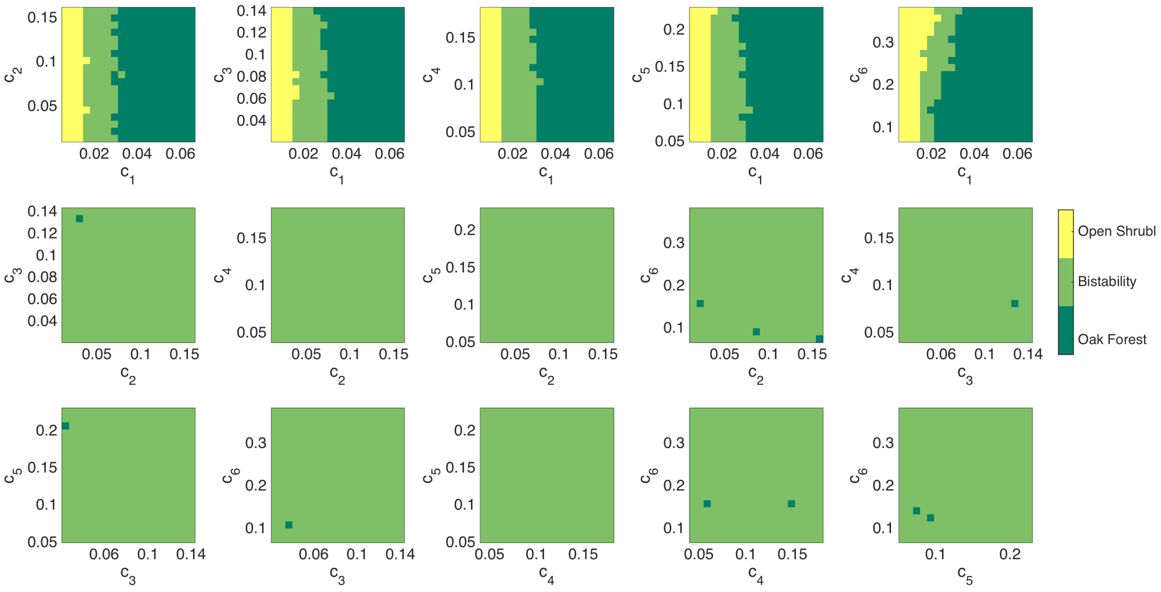

Fig. S 12 Possible states achieved by the system in different areas of the parameter space of the colonization rates. Each panel represents the final state of the system within three categories: "Oak Forest" (more than b_{thr}= 25% oak cover), "Open shrubl" (less than 25% oak cover and more than 25% seeders and/or grass), and "Bistability" (for those cells where both final states are possible). The states are represented as a function of two different colonization rates (between c_1 and c_6; in y^{-1}). This figure is related to panels A to D in Fig. 3, where bistability was observed (r_1=0.60 and flammability three times above baseline). The bimodal area clearly appears within a certain range of oak and grass colonization rates, while for even lower c_1 values only shrublands can persist. This figure is qualitatively unchanged if different values of b_{thr} are used (e.g. b_{thr}= 50%, etc).

Note S5 Short-term experiments

S5.1 Details of short-term experiments

For the short-term experiments with constant aridity (as in Fig. 4 main text), we report here: the initial conditions, the probability distribution of oak and shrub+grass cover after 80-100 yr in runs initialized as an oak forest and as a shrubland with grasses (with the same parameters as in Fig. 4, but different initial conditions). Finally, for all the 48 aridity parameter sets, we report some characteristics of the probability distribution.

Tab. S 12 Sets of initial conditions for the short-term simulations

Initial conditions	*Figure*	$b_{1,0}$	$b_{2,0}$	$b_{3,0}$	$b_{4,0}$	$b_{5,0}$	$b_{6,0}$
Oak forest	Fig. S13	0.89	0.01	0.01	0.01	0.01	0.01
Shrubland with grasses	Fig. S14	0.01	0.01	0.15	0.15	0.15	0.15
Mixed "successional community"	Fig. 4	0.15	0.15	0.15	0.15	0.15	0.15

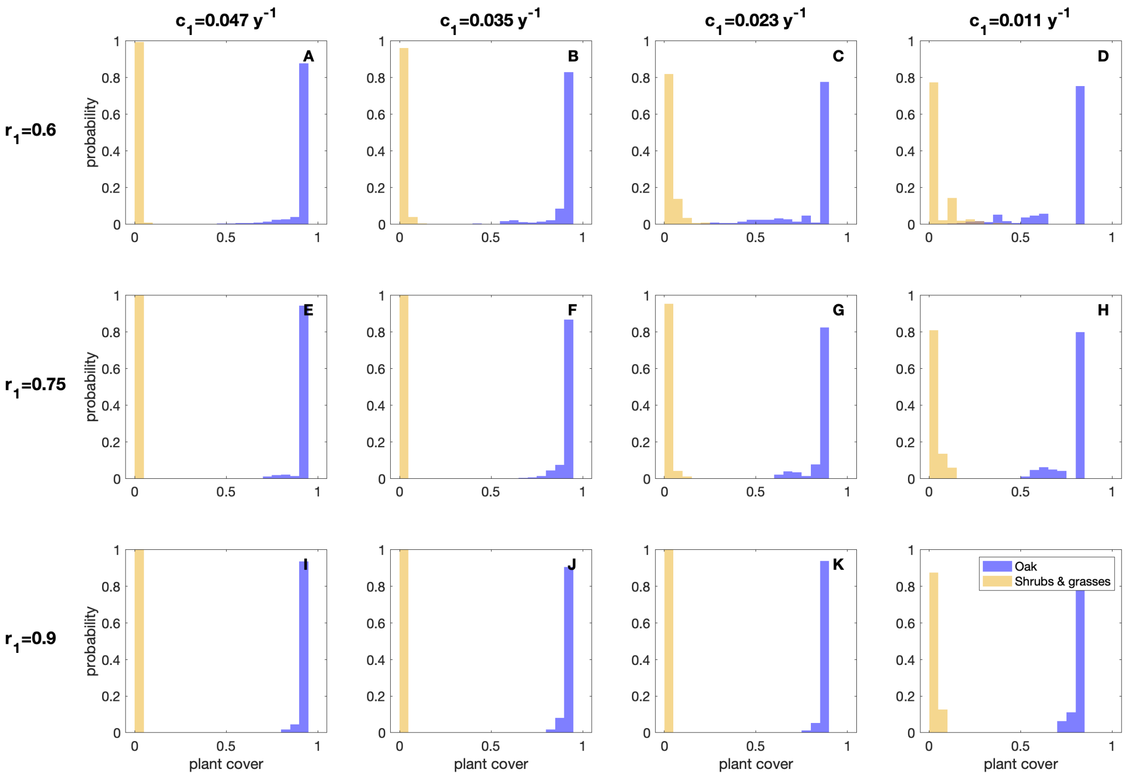

Fig. S 13 Probability distribution of oak cover (blue) and shrubs + grass cover (yellow) after 80-100 yr across the 100 runs for different values of the parameters r_1 and c_1 representing harsher aridity conditions when moving towards the right and upward in the figure. The system was initialized with an oak forest (Table S12). Flammability was 1.5 times the baseline value.

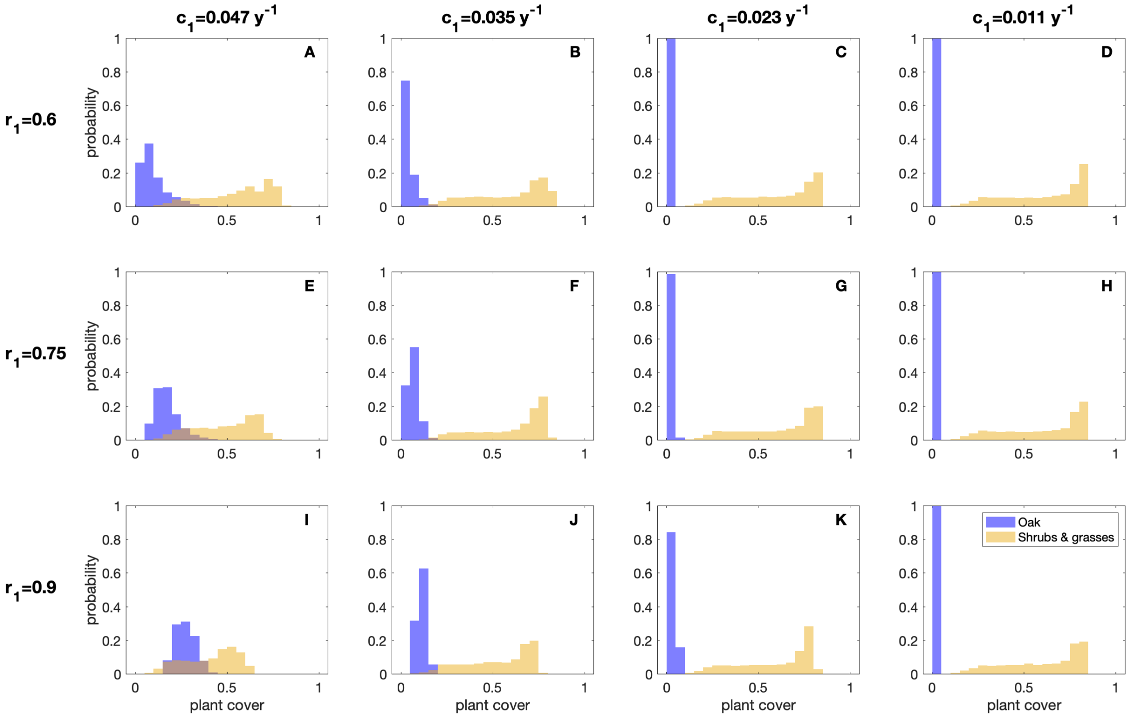

Fig. S 14 Probability distribution of oak cover (blue) and shrubs + grass cover (yellow) after 80-100 yr across the 100 runs for different values of the parameters r_1 and c_1, representing harsher aridity conditions when moving towards the right and upward in the figure. The system was initialised as a shrubland with grasses (Table S12). Flammability was 1.5 times the baseline value.

Tab. S 13 Probability *P* that current oak forests would decay within 100 yr (b_1 below 50% or 65% cover), or would grow above 30% cover if starting from shrubland or mixed successional communities, for the 48 aridity scenarios (given by the c_1, r_1, and flammability multiplication factors with respect to the baseline, Table 2 main text). The orange lines correspond to data specifically mentioned in the main text.

c_1 (yr^{-1})	r_1	flam.	In. c.: mixed successional			In. cond.: oak forest			In. cond: shrubs & grasses		
			b_1 min	b_1 max	P(b_1>0.30)	b_1 min	P(b_1 ≤0.50)	P(0.50< b_1≤0.65)	b_1 min	b_1 max	P(b_1 >0.30)
0.047	0.90	x 1	0.71	0.89	100%	0.85	0%	0%	0.15	0.45	46%
		x 1.2	0.66	0.89	100%	0.84	0%	0%	0.15	0.45	41%
		x 1.5	0.68	0.89	100%	0.77	0%	0%	0.14	0.43	31%
		x 3	0.64	0.89	100%	0.79	0%	0%	0.11	0.40	8%
	0.75	x 1	0.51	0.89	100%	0.71	0%	0%	0.06	0.45	18%
		x 1.2	0.31	0.89	100%	0.71	0%	0%	0.04	0.45	11%
		x 1.5	0.30	0.89	100%	0.71	0%	0%	0.04	0.45	5%
		x 3	0.18	0.89	99%	0.61	0%	0.2%	0.02	0.30	0%
	0.60	x 1	0.23	0.89	98%	0.41	0%	1%	0.02	0.45	5%
		x 1.2	0.10	0.89	97%	0.42	0%	2%	0.02	0.45	4%
		x 1.5	0.13	0.89	93%	0.43	0%	1%	0.01	0.45	2%
		x 3	0.05	0.89	60%	0.36	2%	7%	0.00	0.31	0.1%
0.035	0.90	x 1	0.53	0.77	100%	0.83	0%	0%	0.06	0.20	0%
		x 1.2	0.52	0.77	100%	0.83	0%	0%	0.05	0.20	0%
		x 1.5	0.48	0.77	100%	0.83	0%	0%	0.05	0.20	0%
		x 3	0.44	0.77	100%	0.81	0%	0%	0.04	0.17	0%
	0.75	x 1	0.28	0.77	100%	0.68	0%	0%	0.03	0.20	0%
		x 1.2	0.25	0.77	99%	0.71	0%	0%	0.02	0.20	0%
		x 1.5	0.22	0.77	97%	0.69	0%	0%	0.02	0.20	0%
		x 3	0.11	0.77	73%	0.57	0%	1%	0.01	0.09	0%
	0.60	x 1	0.05	0.77	85%	0.53	0%	2%	0.005	0.20	0%
		x 1.2	0.07	0.77	79%	0.53	0%	5%	0.01	0.20	0%
		x 1.5	0.04	0.77	59%	0.39	0%	3%	0.002	0.20	0%
		x 3	0.01	0.56	14%	0.24	4%	7%	0.001	0.05	0%
0.023	0.90	x 1	0.31	0.54	100%	0.80	0%	0%	0.03	0.07	0%
		x 1.2	0.30	0.54	100%	0.78	0%	0%	0.03	0.07	0%
		x 1.5	0.25	0.54	98%	0.77	0%	0%	0.02	0.07	0%
		x 3	0.24	0.54	91%	0.72	0%	0%	0.02	0.06	0%
	0.75	x 1	0.13	0.54	70%	0.51	0%	1%	0.01	0.07	0%
		x 1.2	0.13	0.54	66%	0.62	0%	1%	0.01	0.07	0%
		x 1.5	0.08	0.54	54%	0.60	0%	2%	0.004	0.06	0%
		x 3	0.04	0.54	5%	0.51	0%	3%	0.002	0.04	0%
	0.60	x 1	0.03	0.54	37%	0.50	0%	1%	0.001	0.07	0%
		x 1.2	0.02	0.54	39%	0.41	2%	6%	0.001	0.04	0%
		x 1.5	0.01	0.54	25%	0.23	6%	8%	0.002	0.05	0%
		x 3	0.00	0.54	6%	0.27	13%	11%	<10^{-3}	0.04	0%
0.011	0.90	x 1	0.13	0.28	0%	0.69	0%	0%	0.010	0.02	0%
		x 1.2	0.14	0.28	0%	0.72	0%	0%	0.009	0.02	0%
		x 1.5	0.12	0.28	0%	0.72	0%	0%	0.009	0.02	0%
		x 3	0.10	0.24	0%	0.65	0%	0%	0.01	0.02	0%
	0.75	x 1	0.05	0.28	0%	0.48	1%	5%	0.004	0.02	0%
		x 1.2	0.02	0.28	0%	0.53	0%	9%	0.003	0.02	0%
		x 1.5	0.04	0.28	0%	0.52	0%	11%	0.002	0.02	0%
		x 3	0.01	0.28	0%	0.29	8%	12%	0.001	0.01	0%
	0.60	x 1	0.01	0.28	0%	0.36	4%	14%	<10^{-3}	0.02	0%
		x 1.2	0.01	0.28	0%	0.22	6%	9%	0.001	0.02	0%
		x 1.5	0.00	0.28	0%	0.14	11%	14%	<10^{-3}	0.02	0%
		x 3	0.00	0.25	0%	0.15	18%	22%	<10^{-3}	0.01	0%

S5.2 Aridity level harshening over time

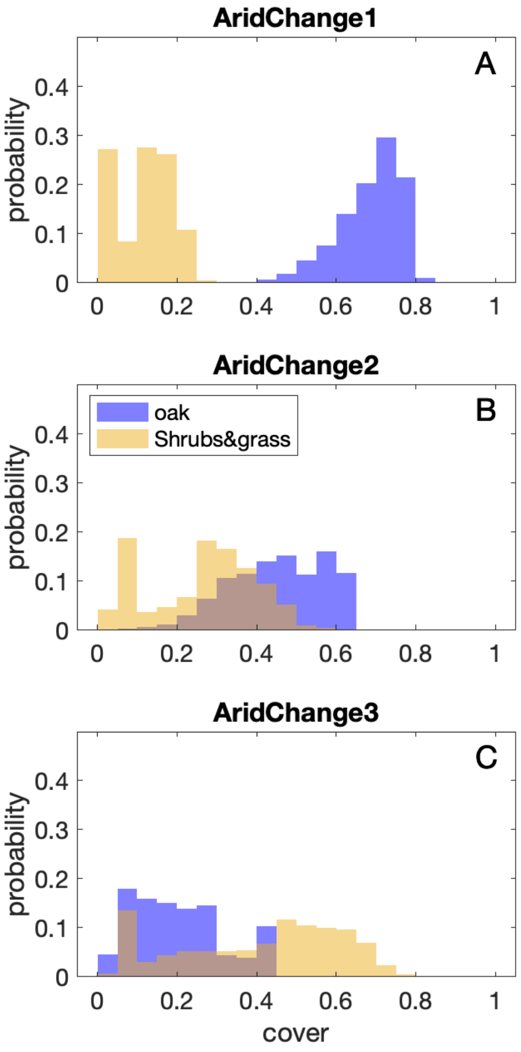

We performed an extra short-term experiment, where aridity increased over time. Specifically, we developed three scenarios of increased aridity (AridChange1-3), by directly decreasing c_1 and r_1 and increasing flammability at three different rates at the same time and during the simulation (see Table S14 below). We assumed aridity to increase linearly in time for the first 50 years, and then to stabilize for the last 50 years. The stabilization is a conservative assumption, which follows some of the least disruptive IPCC scenarios (e.g. Representative Concentration Pathways RCP 2.5, 4.5 and partly 6.0; van Vuuren *et al.*, 2011; Intergovernmental Panel on Climate Change, 2014). We performed 200 runs for each scenario, all starting with a mixed successional community as an initial condition. Again, the results show that the probability of oak forest to grow in the coming century is much reduced by increased aridity levels (see Fig. S15). Thus, the simultaneous increase in aridity of the three parameters did not affect the qualitative results in any major way.

Fig. S 15 Probability distribution of oak cover (blue) and shrubs + grass cover (yellow) after 80-100 yr across the 200 runs for the three aridity scenarios (see below Tab. S14). The system was initialized as a mixed successional community. From top to bottom, sharper aridity increases led to lower probability that a forest would develop in 100 yr (similarly to the experiment described in the main, Fig. 4).

Tab. S 14 Changes in flammability (multiplication factor with respect to baseline values, Table 2 main text), oak colonization rate (c_1) and oak resprouting capacity (r_1) in the three "AridChange" scenarios. Aridity increased in time and affected the three parameters simultaneously, with a. linear increase for the first 50 years of simulation and then a stabilization for the last 50 years.

Scenario	c_1	r_1	*flammab.*	b_1 *min*	b_1 *max*	*probability of* b_1*>0.30*
AridChange1	from 0.047 to 0.035 yr^{-1}	from 0.9 to 0.8	x 1 to x 1.2	0.40	0.80	100%
AridChange2	from 0.047 to 0.023 yr^{-1}	from 0.9 to 0.7	x 1 to x 1.5	0.08	0.64	89%
AridChange3	from 0.047 to 0.011 yr^{-1}	from 0.9 to 0.6	x 1 to x 3	0.02	0.43	18%

References

Akaike H. 1974. A New Look at the Statistical Model Identification. *IEEE Transactions on Automatic Control* **19**: 716–723.

Baquedano FJ, Castillo FJ. 2007. Drought tolerance in the Mediterranean species *Quercus coccifera*, *Quercus ilex*, *Pinus halepensis*, and *Juniperus phoenicea*. *Photosynthetica* **45**: 229–238.

Baudena M, Bevilacqua I, Canone D, Ferraris S, Previati M, Provenzale A. 2012. Soil water dynamics at a midlatitude test site: Field measurements and box modeling approaches. *Journal of Hydrology* **414–415**: 329–340.

Canadell J, Jackson RB, Ehleringer JR, Mooney HA, Sala OE, Schulze E-D. 1996. Maximum rooting depth of vegetation types at the global scale. *Oecologia* **108**: 583–595.

Cardoso MF, Salcedo RL, Feyo de Azevedo S. 1996. The Simplex simulated annealing approach to continuous non-linear optimization. *Computers & chemical Engineering* **2**: 1065–1080.

Climent J, Prada MA, Calama R, Chambel MR, de Ron DS, Alía R. 2008. To grow or to seed: ecotypic variation in reproductive allocation and cone production by young female Aleppo pine (*Pinus halepensis* , Pinaceae). *American Journal of Botany* **95**: 833–842.

Daskalakou EN, Thanos CA. 1996. Aleppo pine (*Pinus halepensis*) postfire regeneration: the role of canopy and soil seed banks. *International Journal of Wildland Fire* **6**: 59–66.

Donckels B. 2009. Optimal experimental design to discriminate among rival dynamic mathematical models. *PhD thesis.* Ghent University, Belgium

Goffe WL, Ferrier GD, Rogers. J. 1994. Global optimization of statistical functions with simulated annealing. *Journal of Econometrics* **60**: 65–99.

Intergovernmental Panel on Climate Change. 2014. *Climate Change 2014 Mitigation of Climate Change*. Cambridge: Cambridge University Press.

Jensen JL. 1906. Sur les fonctions convexes et les inégualités entre les valeurs moyennes. *Acta Mathematica* **30**: 175–193.

Kirkpatrick S, Gelatt CD, Vecchi MP. 1983. Optimization by Simulated Annealing. *Science* **220**: 671–680.

Martínez-Ferri E, Balaguer L, Valladares F, Chico JM, Manrique E. 2000. Energy dissipation in drought-avoiding and drought-tolerant tree species at midday during the Mediterranean summer. *Tree Physiology* **20**: 131–138.

Martínez-Sánchez JJ, Ferrandis P, de las Heras J, Herranz JM. 1999. Effect of burnt wood removal on the natural regeneration of *Pinus halepensis* after fire in a pine forest in Tus valley (SE Spain). *Forest Ecology and Management* **123**: 1–10.

Moya-Delgado S. 2017. Dinámica temporal del esfuerzo reproductivo post-fuego de tres especies germinadoras obligadas. *Master Thesis*. University of Alicante, Spain.

Padilla BA. 1997. Colonización vegetal en campos de cultivo abandonados en la provincia de Alicante. *PhD Thesis*. University of Alicante, Spain.

Paula S, Arianoutsou M, Kazanis D, ÇTavsanoglu, Lloret F, Buhk C, Ojeda F, Luna B, Moreno J, Rodrigo A, *et al.* 2009. Fire-related traits for plant species of the Mediterranean

Basin. *Ecology* **90**: 1420.

Pausas JG. 1999. Response of plant functional types to changes in the fire regime in Mediterranean ecosystems : A simulation approach. *Journal of Vegetation Science* **10**: 717–722.

Pausas JG, Ouadah N, Ferran A, Gimeno T, Vallejo R. 2003. Fire severity and seedling establishment in *Pinus halepensis* woodlands, eastern Iberian Peninsula. *Plant Ecology* **169**: 205–213.

Pausas JG, Pratt RB, Keeley JE, Jacobsen AL, Ramirez AR, Vilagrosa A, Paula S, Kaneakua-Pia IN, Davis SD. 2016. Towards understanding resprouting at the global scale. *New Phytologist* **209**: 945–954.

Peña J. 2007. Efectos ecológicos de los cambios de coberturas y usos del suelo en la Marina Baixa (Alicante). *PhD Thesis*. University of Alicante, Spain.

Rambal S. 1984. Water balance and pattern of root water uptake by a *Quercus coccifera* L. evergreen scrub. *Oecologia* **62**: 18–25.

Rodriguez-Aizpeolea J, Pérez-Badia R, Cerda-Bolinches A. 1991. Colonizacion vegetal y produccion de escorrentia en bancales abandonados: Vall de Gallinera, Alacant. *Cuaternario y Geomorfología* **5**: 119–129.

Santana VM, Alday JG, Baeza MJ. 2014. Effects of fire regime shift in Mediterranean Basin ecosystems: changes in soil seed bank composition among functional types. *Plant Ecology* **215**: 555–566.

Santana VM, Baeza MJ, Blanes MC, Alicante U De. 2013a. Clarifying the role of fire heat and daily temperature fluctuations as germination cues for Mediterranean Basin obligate seeders. *Annals of Botany* **111**: 127–134.

Santana VM, Baeza MJ, Maestre FT. 2012. Seedling establishment along post-fire succession in Mediterranean shrublands dominated by obligate seeders. *Acta Oecologica* **39**: 51–60.

Santana VM, Baeza MJ, Marrs RH. 2013b. Response of woody and herbaceous fuel to repeated fires in Mediterranean gorse shrublands. *International Journal of Wildland Fire* **22**: 508–514.

Santana VM, Baeza MJ, Marrs RH, Vallejo VR. 2010. Old-field secondary succession in SE Spain: can fire divert it? *Plant Ecology* **211**: 337–349.

Tapias R, Gil L, Fuentes-Utrilla P, Pardos JA. 2001. Canopy seed banks in Mediterranean pines of south-eastern Spain: a comparison between *Pinus halepensis* Mill., *P. pinaster* Ait., *P. nigra* Arn. and *P. pinea* L. *Journal of Ecology* **89**: 629–638.

Tatoni T. 1992. Evolution post-culturale des agrosystèmes de terrasses en Provence calcaire. Phytoécologie et impact humain. *PhD thesis*. University of Alicante, Spain.

Tavşanoğlu Ç, Pausas JG. 2018. A functional trait database for Mediterranean Basin plants. *Scientific Data* **5**: 180135.

Tchuinté Tamen A, Dumont Y, Tewa JJ, Bowong S, Couteron P. 2016. Tree-grass interaction dynamics and pulsed fires: Mathematical and numerical studies. *Applied Mathematical Modelling* **40**: 6165–6197.

Tchuinté Tamen A, Dumont Y, Tewa JJ, Bowong S, Couteron P. 2017. A minimalistic model of tree–grass interactions using impulsive differential equations and non-linear feedback

functions of grass biomass onto fire-induced tree mortality. *Mathematics and Computers in Simulation* **133**: 265–297.

Tilman D. 1994. Competition and biodiversity in spatially-structured habitats. *Ecology* **75**: 2–16.

UNEP. 1992. *World Atlas of Desertification* (N Middleton and DSG Thomas, Eds.). London: Edward Arnold.

Verkaik I, Espelta JM. 2006. Post-fire regeneration thinning, cone production, serotiny and regeneration age in *Pinus halepensis*. *Forest Ecology and Management* **231**: 155–163.

van Vuuren DP, Edmonds J, Kainuma M, Riahi K, Thomson A, Hibbard K, Hurtt GC, Kram T, Krey V, Lamarque J-F, *et al.* 2011. The representative concentration pathways: an overview. *Climatic Change* **109**: 5–31.

Yatat V, Couteron P, Tewa JJ, Bowong S, Dumont Y. 2017. An impulsive modelling framework of fire occurrence in a size-structured model of tree–grass interactions for savanna ecosystems. *Journal of Mathematical Biology* **74**: 1425–1482.

Zomer R, Trabucco A, Bossio DA, Verchot L V. 2008. Climate Change Mitigation: A Spatial Analysis of Global Land Suitability for Clean Development Mechanism Afforestation and Reforestation. *Agric. Ecosystems and Envir.* **126**: 67–80.
